# Supplementary material for: Association between non-HDLC and 1-year prognosis in patients with spontaneous intracerebral haemorrhage: a prospective cohort study from 13 hospitals in Beijing
Source: BMJ Open. 2022 Nov 2;12(11):e061241. doi: 10.1136/bmjopen-2022-061241 (PMC9639077; doi:10.1136/bmjopen-2022-061241)
Supplement: Supplementary data [file bmjopen-2022-061241supp001.pdf]

Supplementary Table 1. Odds ratios and 95% CI for 1-year poor outcome (mRS ≥3) according to non-HDLC quartiles.

	non-HDLC quartiles				Continuous scale	*P* for trend
	Q1	Q2	Q3	Q4		
1-year poor outcome, n (%)	71 (43.3)	58 (34.5)	54 (32.3)	40 (24.0)		
Univariate analysis	Ref.	0.69 (0.44, 1.08)	0.63 (0.40, 0.98)	0.41 (0.26, 0.66)	0.76 (0.66, 0.88)	<0.001
Multivariate analysis						
Model 1	Ref.	0.80 (0.50, 1.29)	0.84 (0.52, 1.36)	0.57 (0.35, 0.95)	0.85 (0.73, 1.00)	0.049
Model 2	Ref.	0.81 (0.44, 1.50)	1.03 (0.56, 1.90)	0.71 (0.37, 1.37)	0.93 (0.76, 1.14)	0.468
Sensitivity analysis	Ref.	0.83 (0.43, 1.60)	1.14 (0.60, 2.18)	0.76 (0.39, 1.51)	0.96 (0.77, 1.18)	0.673

Data are OR (95% CI) unless otherwise stated.

Model 1 adjusted for age and sex.

Model 2 adjusted for variates in model 1 plus prior mRS scale (<3 or ≥3) history of ICH, glucose on admission, WBC on admission, baseline hematoma volume, hematoma location, time from onset to initial non-contrast CT, GCS score at admission, systolic blood pressure.

Sensitivity analysis was performed in ICH patients without statin use after admission (n=589), and adjusted for variates in model 2.
