# Supplementary material for: Association between non-HDLC and 1-year prognosis in patients with spontaneous intracerebral haemorrhage: a prospective cohort study from 13 hospitals in Beijing
Source: BMJ Open. 2022 Nov 2;12(11):e061241. doi: 10.1136/bmjopen-2022-061241 (PMC9639077; doi:10.1136/bmjopen-2022-061241)
Supplement: Author's manuscript [file bmjopen-2022-061241.draft_revisions.pdf]

BMJ Open

BMJ Open is committed to open peer review. As part of this commitment we make the peer review history of every article we publish publicly available.

When an article is published we post the peer reviewers' comments and the authors' responses online. We also post the versions of the paper that were used during peer review. These are the versions that the peer review comments apply to.

The versions of the paper that follow are the versions that were submitted during the peer review process. They are not the versions of record or the final published versions. They should not be cited or distributed as the published version of this manuscript.

BMJ Open is an open access journal and the full, final, typeset and author-corrected version of record of the manuscript is available on our site with no access controls, subscription charges or pay-per-view fees (http://bmjopen.bmj.com).

If you have any questions on BMJ Open's open peer review process please email info.bmjopen@bmj.com

BMJ Open

Association between non-HDLC and 1-year prognosis in patients with spontaneous intracerebral hemorrhage: a prospective cohort study

Journal:	*BMJ Open*
Manuscript ID	bmjopen-2022-061241
Article Type:	Original research
Date Submitted by the Author:	20-Jan-2022
Complete List of Authors:	Wang, Yu; Beijing Tiantan Hospital,<br>Wu, Jianwei; Beijing Tiantan Hospital<br>Wang, Anxin; Beijing Tiantan Hospital<br>Jiang, Ruixuan; Beijing Tiantan Hospital, Department of neurology<br>Wang, Wenjuan; Beijing Tiantan Hospital, Capital Medical University, Department of Neurology<br>Zhao, Xingquan; Beijing Tiantan Hospital, Neurology
Keywords:	Stroke < NEUROLOGY, Lipid disorders < DIABETES & ENDOCRINOLOGY, Risk management < HEALTH SERVICES ADMINISTRATION & MANAGEMENT

SCHOLARONE™
Manuscripts

I, the Submitting Author has the right to grant and does grant on behalf of all authors of the Work (as defined in the below author licence), an exclusive licence and/or a non-exclusive licence for contributions from authors who are: i) UK Crown employees; ii) where BMJ has agreed a CC-BY licence shall apply, and/or iii) in accordance with the terms applicable for US Federal Government officers or employees acting as part of their official duties; on a worldwide, perpetual, irrevocable, royalty-free basis to BMJ Publishing Group Ltd ("BMJ") its licensees and where the relevant Journal is co-owned by BMJ to the co-owners of the Journal, to publish the Work in this journal and any other BMJ products and to exploit all rights, as set out in our licence.

The Submitting Author accepts and understands that any supply made under these terms is made by BMJ to the Submitting Author unless you are acting as an employee on behalf of your employer or a postgraduate student of an affiliated institution which is paying any applicable article publishing charge ("APC") for Open Access articles. Where the Submitting Author wishes to make the Work available on an Open Access basis (and intends to pay the relevant APC), the terms of reuse of such Open Access shall be governed by a Creative Commons licence – details of these licences and which Creative Commons licence will apply to this Work are set out in our licence referred to above.

Other than as permitted in any relevant BMJ Author's Self Archiving Policies, I confirm this Work has not been accepted for publication elsewhere, is not being considered for publication elsewhere and does not duplicate material already published. I confirm all authors consent to publication of this Work and authorise the granting of this licence.

For peer review only

Association between non-HDLC and 1-year prognosis in patients with spontaneous intracerebral hemorrhage: a prospective cohort study

Yu Wang,[1,2*] Jianwei Wu,[1,2*] Anxin Wang,[1,2] Ruixuan Jiang,[1,2] Wenjuan Wang,[1,2] Xingquan Zhao[1,2,3#]

[1] China National Clinical Research Center for Neurological Diseases, Beijing, China

[2] Department of Neurology, Beijing Tiantan Hospital, Capital Medical University, Beijing, China

[3] Research Unit of Artificial Intelligence in Cerebrovascular Disease, Chinese Academy of Medical Sciences, Beijing, China

*YW and JW contributed equally and share the first authorship.

The authors declare that they have no conflicts of interest.

#Correspondence to: Xingquan Zhao (Orcid ID: 0000-0001-8345-5147)

Address: No. 119 South 4th Ring West Road, Fengtai District, Beijing 100070, China.

Telephone: 086-10-59975549 Fax: 086-10-59975549

Email: zxq@vip.163.com

Keywords: non-HDLC, intracerebral hemorrhage, prognosis, risk factors, mRS

Total number of tables and figures: 2 tables and 2 figures.

Word Count: 3847

For peer review only

For peer review only - http://bmjopen.bmj.com/site/about/guidelines.xhtml

ABSTRACT

Objectives: Previous studies suggested an inverse association between lipoprotein cholesterols and bleeding risk, while limited data was available about the predictive value of lipoproteins on intracerebral hemorrhage (ICH). Our recent research series showed that non-high-density lipoprotein cholesterol (non-HDLC) was an independent predictor of 3-month poor prognosis in ICH patients, we thus aimed to further investigate the association between non-HDLC levels and 1-year functional outcomes after ICH.

Design: Prospective multicenter cohort study.

Setting: 13 hospitals in Beijing, China.

Participants: A total of 666 ICH patients were included between December 2014 and September 2016.

Methods: Non-HDLC was calculated by subtracting HDL-C from TC**.** Patients were then grouped by non-HDLC levels into three categories: <3.4mmol/L, 3.4-4.2mmol/L, and ≥4.2mmol/L. Both the univariate and multivariate logistic regressions were used to assess the association between non-HDLC levels and 1-year unfavorable functional outcomes (modified Rankin Scale ≥3) in ICH patients. Moreover, sensitivity analysis was performed in ICH patients without statin use after admission.

Results: There were 33.5% (223/666) ICH patients identified with unfavorable functional outcomes at 1-year follow-up. In the univariate analysis, patients who achieved non-HDLC levels above 4.2 mmol/L had a 49% decreased risk of 1-year poor prognosis (OR 0.51, 95% CI 0.33-0.81). However, non-HDLC did not retain its independent prognostic value in multivariate analysis, the fully adjusted OR values were 1.00 (reference), 0.99 (0.59-1.67), and 0.88 (0.48-1.62) from the lowest to the highest non-HDLC group Moreover, statin use after ICH onset made no difference to the long-term prognosis.

Conclusions: Non-HDLC was not an independent predictor for 1-year functional outcome in ICH patients, irrespective of post-stroke statin use. The predictive value of well-recognized confounding factors was

For peer review only - http://bmjopen.bmj.com/site/about/guidelines.xhtml

more dominant than non-HDLC on long-term prognosis.

Strengths and limitations of this study

- A multicenter, prospective, cohort study included 666 ICH patients from a total of 13 hospitals in Beijing.
- Our study filled the vacancy about the association between non-HDLC and 1-year functional outcomes, simultaneously shed light on the diverse impacts of non-HDLC on short-term and long-term prognosis in ICH patients.
- Sensitivity analysis was performed to evaluate the association between non-HDLC and 1-year functional outcomes in ICH patients with post-stroke statin use.
- Factors including radiological information or antithrombotic treatment may affect the results.

For peer review only

For peer review only - http://bmjopen.bmj.com/site/about/guidelines.xhtml

INTRODUCTION

Intracerebral hemorrhage (ICH) is the second most common subtype of stroke, leading to severe disability and mortality.[1] Based on the nationally representative stroke survey in China published recently, ICH accounts for 25% of all strokes with an overall age-standardized incidence of 66.2 per 100,000 person-years.[2] Despite rapid advances in medicine, the management of ICH remains supportive without significant breakthroughs.[3] Approximately 30-48% of ICH patients died within one month in low- to middle-income countries and only 12-39% of survivors could achieve long-term functional independence.[1, 4]

The conventional view on lipid-lowering targets goes "the lower, the better" in patients with atherosclerotic cardiovascular disease. However, previous epidemiology studies suggested an inverse association between lipoprotein cholesterols and ICH risk, hematoma expansion, and mortality.[5, 6] Much remains to be discussed on the predictive value of lipoproteins on ICH. Our recent research series showed that low serum lipid levels were independent predictors of 3-month poor prognosis in ICH patients, and non-high-density lipoprotein cholesterol (non-HDLC) was the optimal parameter with high specificity.[7, 8] However, the literature has scant information regarding the association between non-HDLC and long-term ICH prognosis.

We thus aimed to investigate the association between serum non-HDLC levels and 1-year functional outcomes after ICH in this prospective cohort study.

METHODS

Study population

The study was conducted in accordance with the Declaration of Helsinki and was approved by

For peer review only - http://bmjopen.bmj.com/site/about/guidelines.xhtml

the Institutional Review Board of the Beijing Tiantan Hospital (KY2014-023-02). All participants or their legal representatives provided written informed consent.

Our study is a multicenter, prospective, cohort study conducted in a total of 13 hospitals, evaluating the medical quality of cerebral hemorrhage on different etiologies in Beijing. From December 2014 to September 2016, 1964 consecutive ICH patients agreed to participate in the study. A total of 1881 patients met the following inclusion criteria: (1) aged 18 years or older, (2) had their first CT scan done within 72h after symptom onset. After excluding 159 secondary ICH patients (caused by trauma, tumor, aneurysm, arteriovenous malformation, coagulopathy, or other causes) and 20 patients diagnosed as primary ventricular hemorrhage, 1702 patients with primary intraparenchymal hemorrhage were included. Moreover, 294 patients underwent surgical procedures (including craniotomy hematoma removal, hematoma puncture, extraventricular drainage, and so on), 15 patients with anticoagulant therapy before symptom onset, 588 patients with missing data on the non-HDLC level, and 139 patients lost to follow-up at 1-year were excluded. Eventually, 666 patients with spontaneous ICH from 13 sites were included (Figure 1).

Baseline information

Demographic information including age, sex, onset to admission time, past medical history (including hypertension, diabetes mellitus, hyperlipidemia, cerebral infarction, and ICH), personal habits (including smoking and drinking status), and medication history (including antiplatelet and statin therapy) of each patient was collected using a standard questionnaire at baseline. Neurological deficits were assessed using the National Institute of Health Stroke Scale (NIHSS)

For peer review only - http://bmjopen.bmj.com/site/about/guidelines.xhtml

and Glasgow Coma Scale (GCS) score by experienced neurologists on admission. Meanwhile, systolic and diastolic blood pressure (BP) were measured. A cranial CT scan was performed on admission and hematoma volume was then calculated as ABC/2 volumetric formula at each site.[9] The location of hematoma was further subdivided into supratentorial and infratentorial regions.

Measurement of non-HDLC levels and other biochemical parameters

Blood samples were drawn from the antecubital vein the next morning after an overnight fast and analyzed within 4h. Total cholesterol (TC) was measured using the end-point test method and high-density lipoprotein cholesterol (HDL-C) was measured using a direct method. Non-HDLC was thus calculated by subtracting HDL-C from TC. Based on the National Lipid Association Recommendations,[10] non-HDLC levels were categorized into five groups: desirable, <3.4mmol/L; above desirable, 3.4-4.2mmol/L; borderline high, 4.2-5.0mmol/L; high, 5.0-5.8 mmol/L; and very high, ≥5.8 mmol/L. Accordingly, we integrated the last three groups into one group (≥4.2mmol/L) due to the limited number of patients.

For other biochemical parameters, random blood glucose was measured via the hexokinase/glucose-6-phosphate dehydrogenase method, serum creatinine was measured through rate reflectance spectrophotometry, white blood cell (WBC) together with platelet count were performed on EDTA with an ADVIA 120 counter (Siemens Healthcare Diagnostics, Saint-Denis, France).

Follow-up information and definition of 1-year ICH prognosis

Patients were followed up at 1-year after ICH onset via telephone interviews. Follow-up

For peer review only - http://bmjopen.bmj.com/site/about/guidelines.xhtml

evaluation was performed by neurologists who were blinded to prognostic factors. 1-year prognosis of patients was evaluated by modified Ranking Scale (mRS) score and categorized as favorable (mRS<3) and unfavorable functional outcome groups (mRS≥3).

Patient and public involvement

No patients were involved.

Statistical analysis

The patients were divided into three groups according to the clinical diagnosis of abnormal non-HDLC levels. Continuous variables were presented as median with interquartile range (IQR), categorical variables were described as count with percentage. The group differences of continuous variables were analyzed using ANOVA or Kruskal-Wallis test as appropriate, and for categorical variables, chi-squared tests were performed. Logistic regression was used to evaluate the association between non-HDLC levels and 1-year prognosis of ICH patients, with the lowest non-HDLC group (<3.4mmol/L) used as the reference. Both the univariate and multivariate analyses were conducted to estimate the odds ratios (ORs) and 95% confidence intervals (CIs). Multiple regression models were run as follows. Model 1 was adjusted for age and sex. Model 2 was adjusted for variates in model 1 plus history of ICH, glucose on admission, WBC on admission, baseline hematoma volume, hematoma location, time from onset to initial non-contrast CT, GCS score at admission, and systolic BP. *P*-values for trend were conducted using the three categories of non-HDLC as ordinal variables in the model. Additionally, sensitivity analysis was performed in ICH patients without statin use after admission (n=589). A 2-sided value of

For peer review only - http://bmjopen.bmj.com/site/about/guidelines.xhtml

$p<0.05$ was considered statistically significant. All statistical analyses were performed using SAS software, version 9.4 (SAS Institute, Cary, NC, USA).

RESULTS

A total of 666 eligible patients were included, with a mean age of 59 years old (ranging from 51 to 68) and 69.1% (460/666) of them were males. Amongst them, 33.5% (223/666) were identified as 1-year poor outcomes, the proportion of which were 38.4%, 30.3%, and 24.2% from <3.4mmol/L group to ≥4.2mmol/L group.

Baseline characteristics

There were significant differences in age, prior statin use, diastolic BP, glucose on admission, WBC on admission, and statin use after admission among the three categories of non-HDLC levels ($p<0.05$, Table 1). Those with higher lipid levels were more likely to be younger, not a prior statin user, having higher diastolic BP and glucose on admission.

Table 1. Baseline characteristics of participants according to non-HDLC levels.

	Total	non-HDLC levels			*P*-value
		<3.4mmol/L	3.4-4.2mmol/L	⩾4.2mmol/L	
n (%)	666	359 (53.9)	175 (26.3)	132 (19.8)	
Age, years	59 (51, 68)	61 (53, 70)	57 (49, 67)	54 (48, 64)	<0.001
Male, n (%)	460 (69.1)	258 (71.9)	120 (68.6)	82 (62.1)	0.116
Onset to admission time, h	4.0 (1.8, 11.9)	3.8 (1.7, 11.1)	4.0 (2.0, 11.0)	4.0 (1.8, 14.7)	0.840
Hypertension, n (%)	479 (71.9)	256 (71.3)	124 (70.9)	99 (75.0)	0.676
Diabetes mellitus, n (%)	106 (15.9)	55 (15.3)	29 (16.6)	22 (16.7)	0.902
Hyperlipidemia, n (%)	68 (10.2)	36 (10.0)	18 (10.3)	14 (10.6)	0.982
History of CI, n (%)	102 (15.3)	58 (16.2)	27 (15.4)	17 (12.9)	0.670
History of ICH, n (%)	20 (3.0)	15 (4.2)	3 (1.7)	2 (1.5)	0.141
Smoking, n (%)	223 (33.5)	127 (35.4)	57 (32.6)	39 (29.6)	0.458
Drinking, n (%)	256 (38.4)	139 (38.7)	69 (39.4)	48 (36.4)	0.850
Prior antiplatelet use, n (%)	110 (16.5)	61 (17.0)	28 (16.0)	21 (15.9)	0.771
Prior statin use, n (%)	44 (6.6)	31 (8.6)	10 (5.7)	3 (2.3)	0.036

For peer review only - http://bmjopen.bmj.com/site/about/guidelines.xhtml

NIHSS score on admission	8 (3, 13)	9 (3, 15)	7 (3, 13)	5 (2, 12)	0.083
GCS score on admission	14 (12, 15)	14 (12, 15)	15 (13, 15)	15 (13, 15)	0.063
SBP on admission, mmHg	160 (149, 183)	160 (150, 180)	160 (145, 183)	162 (150, 183)	0.564
DBP on admission, mmHg	95 (83, 105)	92 (80, 102)	96 (85, 106)	97 (85, 109)	0.024
Glucose on admission, mmol/L	6.9 (5.9, 8.4)	6.6 (5.8, 8.1)	7.0 (5.9, 8.6)	7.1 (6.0, 9.3)	0.032
WBC on admission, 10^9/L	8.4 (6.6, 10.9)	8.1 (6.3, 10.7)	9.1 (7.0, 11.7)	7.1 (6.0, 9.3)	0.007
Platelets on admission, 10^9/L	212 (175, 252)	202 (164, 238)	218 (180, 259)	230 (192, 265)	<0.001
Creatinine on admission, μmol/L	64.0 (53.0, 77.3)	64.6 (54.0, 76.4)	65.0 (52.3, 79.0)	62.0 (50.1, 76.0)	0.223
Statin use after admission, n (%)	77 (11.6)	19 (5.3)	30 (17.1)	28 (21.2)	<0.001
Infections, n (%)	136 (20.4)	77 (21.5)	39 (22.3)	20 (15.2)	0.239
Time from onset to initial NCCT, h	5.2 (2.3, 16.7)	5.2 (2.2, 14.8)	5.1 (2.3, 19.6)	4.8 (2.3, 19.4)	0.738
Baseline hematoma volume, ml	10.5 (5.0, 23.4)	10.7 (5.0, 25.0)	10.4 (5.5, 23.1)	10.0 (4.9, 16.8)	0.379
Hematoma location					0.251
Supratentorial, n (%)	599 (89.7)	327 (91.2)	155 (88.2)	117 (87.5)	
Infratentorial, n (%)	67 (10.3)	31 (8.8)	23 (11.8)	16 (12.5)	
ICH score	0 (0, 1)	0 (0, 1)	0 (0, 1)	0 (0, 1)	0.447

Values are (%) for categorical variables and median (IQR) for continuous variables.

CI, cerebral infarction; ICH, intracerebral hemorrhage; NIHSS, National Institutes of Health Stroke Scale; GCS, Glasgow Coma Scale; SBP, systolic blood pressure; DBP, diastolic blood pressure; WBC, white blood cells; NCCT, non-contrast CT.

For peer review only

Correlation between baseline non-HDLC and 1-year prognosis in ICH patients

In the univariate analysis, higher non-HDLC levels were significantly associated with decreased risk of 1-year poor outcome (*p*=0.002). Patients who achieved non-HDLC above 4.2mmol/L had a 49% lower risk of poor functional outcome at 1 year (OR 0.51, 95% CI 0.33-0.81). While no statistical difference was retained after adjusting for age, sex, and potential confounding factors (*p*>0.05). In the fully adjusted model (Model 2), the OR values were 1.00 (reference), 0.99 (0.59-1.67), and 0.88 (0.48-1.62) from the lowest to the highest non-HDLC group. Moreover, the results maintained consistency in sensitivity analysis among patients without statin use after admission (*p*=0.791, Table 2).

Table 2. Odds ratios and 95% CI for 1-year poor outcome (mRS ≥3) according to non-HDLC levels.

	non-HDLC levels			Continuous scale	*P* for trend
	<3.4mmol/L	3.4-4.2mmol/L	⩾4.2mmol/L		
1-year poor outcome, n (%)	138 (38.4)	53 (30.3)	32 (24.2)		
Univariate analysis	Ref.	0.70 (0.47, 1.02)	0.51 (0.33, 0.81)	0.71 (0.58, 0.88)	0.002

For peer review only - http://bmjopen.bmj.com/site/about/guidelines.xhtml

Multivariate analysis					
Model 1	Ref.	0.82 (0.54, 1.23)	0.66 (0.41, 1.06)	0.81 (0.65, 1.02)	0.075
Model 2	Ref.	0.99 (0.59, 1.67)	0.88 (0.48, 1.62)	0.95 (0.71, 1.27)	0.710
Sensitivity analysis	Ref.	1.16 (0.67, 2.00)	0.86 (0.46, 1.62)	0.96 (0.71, 1.30)	0.791

Data are OR (95% CI) unless otherwise stated.

Model 1 adjusted for age and sex.

Model 2 adjusted for variates in model 1 plus history of ICH, glucose on admission, WBC on admission, baseline hematoma volume, hematoma location, time from onset to initial non-contrast CT, GCS score at admission, systolic blood pressure.

Sensitivity analysis was performed in ICH patients without statin use after admission (n=589), and adjusted for variates in model 2.

For peer review only

Notably, age, the history of ICH, and baseline hematoma volume were positively associated with 1-year poor prognosis in the multivariate analysis. Whereas, higher GCS score at admission was an independent predictor of favorable outcomes. Additional detailed information was given in Figure 2.

DISCUSSION

This study provided evidence on the association between non-HDLC levels and long-term functional outcomes in ICH patients. Although non-HDLC was a significant 1-year predictor in univariate analysis, it did not retain its independent prognostic value in multivariate analysis. Moreover, statin use after ICH onset made no difference to the long-term prognosis.

In our study, the prevalence of 1-year functional independence in ICH patients was 66.5% (443/666), far outweighing the data previously reported.[4] According to the inclusion and exclusion criteria, severe cases who underwent surgical treatment or lost to follow-up were not enrolled. It is noteworthy that per 1 mmol/L increment in non-HDLC yielded a 29% decreased risk of 1-year poor prognosis (crude OR 0.71, 95% CI 0.58-0.88). However, contrary to our previous research finding of the independent role of non-HDLC on short-term functional outcomes,[7] the

For peer review only - http://bmjopen.bmj.com/site/about/guidelines.xhtml

results of this study showed that age, the history of ICH, baseline hematoma volume, admission GCS score, rather than non-HDLC level, were independent predictors for long-term functional outcomes in ICH patients. The validated predictors mentioned above kept high conformity with the items in ICH Functional Outcome Score, an effective prognostic model for 1-year poor functional outcomes after ICH,[11] whereas the absolute magnitude effect of low non-HDLC level on ICH prognosis was likely to be small and overshadowed with time.

It was reported that low levels of LDL-C and TC were associated with hematoma expansion.[12, 13] As containing all the atherogenic lipoproteins, non-HDLC was served as the preferred target of lipid-lowering therapy.[14] The potential mechanisms regarding the association between hypolipidemia and hematoma expansion, including impaired endothelial integrity,[15] necrotic medial smooth muscle cells,[16] increased erythrocyte fragility,[17] inhibited platelet aggregation,[18] and the resultant incident cerebral microbleeds.[19] Despite the theoretical basis, our study failed to show an independent correlation between non-HDLC levels and 1-year functional outcomes in ICH patients. The secondary injury caused by low levels of lipoproteins in ICH patients was associated with short-term prognosis (30-day, 3-month),[20, 21] while its impact on long-term prognosis (1-year) was negative, which merits further investigation due to the limited sample size and incomplete neuroimaging data on hematoma expansion in our study.

Statin treatment is another major concern,[22] there were respectively 6.6% (44/666) and 11.6% (77/666) patients with pre- and post-stroke statin use in our study. Two recent meta-analyses concluded that there was no evidence to suggest pre-stroke statin therapy may increase bleeding risk in the context of ICH.[23, 24] Whether to start, continue, or stop statin treatment in ICH patients has aroused great concern, we thus conducted a sensitivity analysis to evaluate the

For peer review only - http://bmjopen.bmj.com/site/about/guidelines.xhtml

effect of statin exposure after admission on ICH prognosis. No significant difference was detected between non-HDLC levels and 1-year prognosis in ICH patients in our study, irrespective of post-stroke statin use. A recent review indicated that statin should be applied after weighing the pros and cons given its pleiotropic as well as lipid-lowering effects.[25] Because of the relatively low stain exposure rate in our study, it is necessary to conduct randomized controlled trials around this topic.

Our study filled the vacancy about the association between non-HDLC and 1-year functional outcomes, simultaneously shed light on the diverse impacts of non-HDLC on short-term and long-term prognosis in ICH patients. Nonetheless, there are still some limitations. First, the follow-up radiological information was unavailable, which makes it difficult to verify the intermediate role of hematoma expansion between non-HDLC and poor prognosis. Secondly, medication therapy regarding antiplatelet or anticoagulation agents were not included in the multivariate analysis, whereas accumulating researches proved that antithrombotic treatment increased the risk of cerebral microbleeds as well as future ICH.[26, 27] Although we collected pre-ictus antiplatelet use, restricted by the small sample size, further research is needed to provide insight into the relationship.

CONCLUSION

In conclusion, non-HDLC was not an independent predictor for 1-year functional outcome in ICH patients, irrespective of post-stroke statin use. The predictive value of well-recognized confounding factors was more dominant than non-HDLC on long-term poor prognosis. Further prospective studies are needed to assess the impact of lower non-HLDC levels on ICH prognosis.

For peer review only - http://bmjopen.bmj.com/site/about/guidelines.xhtml

For peer review only

Author affiliations

1 China National Clinical Research Center for Neurological Diseases, Beijing, China

2 Department of Neurology, Beijing Tiantan Hospital, Capital Medical University, Beijing, China

3 Research Unit of Artificial Intelligence in Cerebrovascular Disease, Chinese Academy of Medical Sciences, Beijing, China

Contributors YW and JW performed the experiments, interpreted the results of statistical analysis, and drafted the manuscript. AW conducted the statistical analysis and interpreted the data. RJ and WW revised the manuscript for intellectual content. XZ had full access to all of the data and take responsibility for the integrity of the data and the accuracy of the data analysis.

Funding Our study was supported by grants from the Chinese Academy of Medical Sciences Innovation Fund for Medical Sciences (2019-I2M-5-029), Beijing Natural Science Foundation (Z200016), Beijing Municipal Committee of Science and Technology (Z201100005620010), and Ministry of Science and Technology of the People's Republic of China (2018YFC1705003).

Competing interests None declared.

Patient consent for publication Not applicable.

Ethics approval The study was approved by the Central Institutional Review Board of Beijing Tiantan Hospital (KY2014-023-02) and written informed consent was obtained.

Provenance and peer review Not commissioned; externally peer reviewed.

For peer review only - http://bmjopen.bmj.com/site/about/guidelines.xhtml

Data sharing statement Some or all datasets generated during and/or analyzed during the current study are not publicly available but are available from the corresponding author on reasonable request.

References

1. An SJ, Kim TJ, Yoon BW. Epidemiology, Risk Factors, and Clinical Features of Intracerebral Hemorrhage: An Update. J Stroke 2017;19:3-10.
2. Wang W, Jiang B, Sun H, et al. Prevalence, Incidence, and Mortality of Stroke in China: Results from a Nationwide Population-Based Survey of 480 687 Adults. Circulation 2017;135:759-771.
3. Hemphill JC 3rd, Greenberg SM, Anderson CS, et al. Guidelines for the Management of Spontaneous Intracerebral Hemorrhage: A Guideline for Healthcare Professionals From the American Heart Association/American Stroke Association. Stroke 2015;46:2032-2060.
4. Feigin VL, Lawes CM, Bennett DA, Barker-Collo SL, Parag V. Worldwide stroke incidence and early case fatality reported in 56 population-based studies: a systematic review. Lancet Neurol 2009;8:355-369.
5. Wang X, Dong Y, Qi X, Huang C, Hou L. Cholesterol levels and risk of hemorrhagic stroke: a systematic review and meta-analysis. Stroke 2013;44:1833-1839.
6. Chang JJ, Katsanos AH, Khorchid Y, et al. Higher low-density lipoprotein cholesterol levels are associated with decreased mortality in patients with intracerebral hemorrhage. Atherosclerosis 2018;269:14-20.
7. Feng H, Wang X, Wang W, Zhao X. Association Between Non-high-density Lipoprotein Cholesterol and 3-Month Prognosis in Patients With Spontaneous Intracerebral Hemorrhage. Front Neurol 2020;11:920.
8. Feng H, Wang X, Wang W, Zhao X. Lipid Levels and 3-Month Prognosis After Spontaneous Intracerebral Hemorrhage in Women. Front Neurol 2021;12:690194.
9. Kothari RU, Brott T, Broderick JP, et al. The ABCs of measuring intracerebral hemorrhage volumes. Stroke 1996;27:1304-1305.
10. Jacobson TA, Ito MK, Maki KC, et al. National lipid association recommendations for patient-centered management of dyslipidemia: part 1--full report. J Clin Lipidol 2015;9:129-169.
11. Ji R, Shen H, Pan Y, et al. A novel risk score to predict 1-year functional outcome after intracerebral hemorrhage and comparison with existing scores. Crit Care 2013;17:R275.
12. Rodriguez-Luna D, Rubiera M, Ribo M, et al. Serum low-density lipoprotein cholesterol level predicts hematoma growth and clinical outcome after acute intracerebral hemorrhage. Stroke 2011;42:2447-2452.
13. Broderick JP, Diringer MN, Hill MD, et al. Determinants of intracerebral hemorrhage growth: an exploratory analysis. Stroke 2007;38:1072-1075.
14. National Cholesterol Education Program Coordinating Committee. Third Report of the National Cholesterol Education Program (NCEP) Expert Panel on Detection, Evaluation, and Treatment of High Blood Cholesterol in Adults (Adult Treatment Panel III) final report. Circulation

For peer review only - http://bmjopen.bmj.com/site/about/guidelines.xhtml

2002;106:3143-3421.

15. Bang OY, Saver JL, Liebeskind DS, et al. Cholesterol level and symptomatic hemorrhagic transformation after ischemic stroke thrombolysis. Neurology 2007;68:737-742.
16. Ooneda G, Yoshida Y, Suzuki K, et al. Smooth muscle cells in the development of plasmatic arterionecrosis, arteriosclerosis, and arterial contraction. Blood Vessels 1978;15:148-156.
17. Yamori Y, Nara Y, Horie R, Ooshima A. Abnormal membrane characteristics of erythrocytes in rat models and men with predisposition to stroke. Clin Exp Hypertens 1980;2:1009-1021.
18. Chui DH, Marotta F, Rao ML, Liu DS, Zhang SC, Ideo C. Cholesterol-rich LDL perfused at physiological LDL-cholesterol concentration induces platelet aggregation and PAF-acetylhydrolase activation. Biomed Pharmacother 1991;45:37-42.
19. Lee SH, Bae HJ, Yoon BW, Kim H, Kim DE, Roh JK. Low concentration of serum total cholesterol is associated with multifocal signal loss lesions on gradient-echo magnetic resonance imaging: analysis of risk factors for multifocal signal loss lesions. Stroke 2002;33:2845-2849.
20. Rodriguez-Luna D, Rubiera M, Ribo M, et al. Ultraearly hematoma growth predicts poor outcome after acute intracerebral hemorrhage. Neurology 2011;77:1599-1604.
21. Sembolini A, Romoli M, Pannacci U, et al. Acute hematoma expansion after spontaneous intracerebral hemorrhage: risk factors and impact on long-term prognosis. Neurol Sci 2020;41:2503-2509.
22. Goldstein LB, Amarenco P, Szarek M, et al. Hemorrhagic stroke in the Stroke Prevention by Aggressive Reduction in Cholesterol Levels study. Neurology 2008;70:2364-2370.
23. Hackam DG, Woodward M, Newby LK, et al. Statins and intracerebral hemorrhage: collaborative systematic review and meta-analysis. Circulation 2011;124:2233-2242.
24. Ziff OJ, Banerjee G, Ambler G, Werring DJ. Statins and the risk of intracerebral haemorrhage in patients with stroke: systematic review and meta-analysis. J Neurol Neurosurg Psychiatry 2019;90:75-83.
25. Endres M, Nolte CH, Scheitz JF. Statin Treatment in Patients With Intracerebral Hemorrhage. Stroke 2018;49:240-246.
26. Qiu J, Ye H, Wang J, Yan J, Wang J, Wang Y. Antiplatelet Therapy, Cerebral Microbleeds, and Intracerebral Hemorrhage: A Meta-Analysis. Stroke 2018;49:1751-1754.
27. Lioutas VA, Goyal N, Katsanos AH, et al. Optimization of risk stratification for anticoagulation-associated intracerebral hemorrhage: net risk estimation. J Neurol 2020;267:1053-1062.

For peer review only

For peer review only - http://bmjopen.bmj.com/site/about/guidelines.xhtml

Figure Legends

Figure 1. Flow chart for selection of study participants.

ICH, intracerebral hemorrhage; non-HDLC, non-high-density lipoprotein cholesterol.

Figure 2. Multivariate predictors of 1-year poor outcome among ICH patients.

Non-HDLC, non-high-density lipoprotein cholesterol; ICH, intracerebral hemorrhage; WBC, white blood cells; NCCT, non-contrast CT; GCS, Glasgow Coma Scale; SBP, systolic blood pressure.

For peer review only

For peer review only - http://bmjopen.bmj.com/site/about/guidelines.xhtml

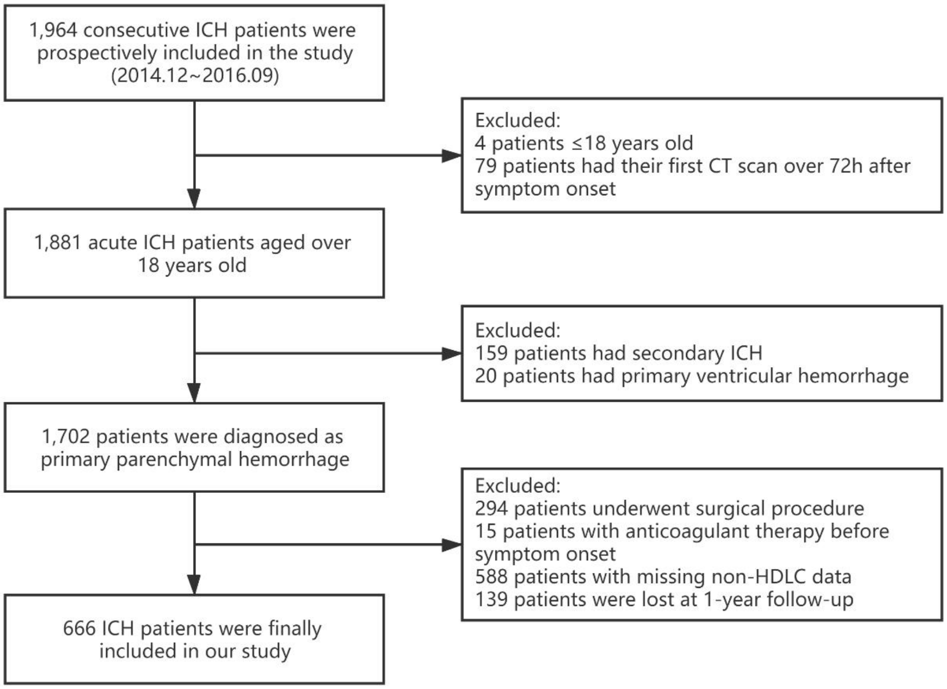

Figure 1. Flow chart for selection of study participants.
ICH, intracerebral hemorrhage; non-HDLC, non-high-density lipoprotein cholesterol.

146x108mm (220 x 220 DPI)

Variables	Poor outcome (mRS ≥3, n=178)	Odds ratio (95% CI)
non-HDLC (vs ≤3.4mmol/L))		
3.4-4.2mmol/L	0.99 (0.59, 1.67)	
≥4.2mmol/L	0.88 (0.48, 1.62)	
Age	1.08 (1.06, 1.10)	
Male	0.83 (0.51, 1.35)	
History of ICH	3.98 (1.32, 5.46)	
Glucose on admission	1.02 (0.95, 1.10)	
WBC on admission	1.01 (0.94, 1.09)	
Platelet on admission	1.00 (0.99, 1.00)	
Baseline hematoma volume	1.02 (1.01, 1.04)	
Infratentorial hemorrhage	0.82 (0.37, 1.82)	
Time from onset to initial NCCT	0.99 (0.98, 1.01)	
GCS score at admission	0.73 (0.66, 0.80)	
SBP	1.00 (0.99, 1.00)	

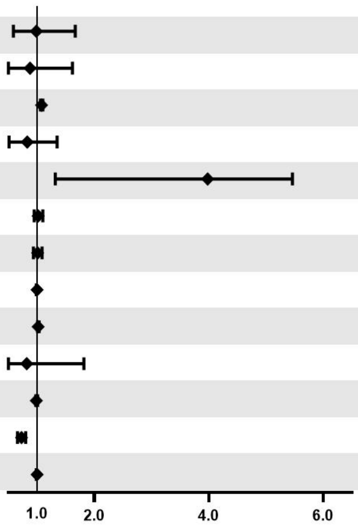

Figure 2. Multivariate predictors of 1-year poor outcome among ICH patients.
Non-HDLC, non-high-density lipoprotein cholesterol; ICH, intracerebral hemorrhage; WBC, white blood cells; NCCT, non-contrast CT; GCS, Glasgow Coma Scale; SBP, systolic blood pressure.

174x108mm (192 x 192 DPI)

STROBE Statement—Checklist of items that should be included in reports of ***cohort studies***

	Item No	Recommendation	Page No
**Title and abstract**	1	(*a*) Indicate the study's design with a commonly used term in the title or the abstract	1
		(*b*) Provide in the abstract an informative and balanced summary of what was done and what was found	2
**Introduction**			
Background/rationale	2	Explain the scientific background and rationale for the investigation being reported	4
Objectives	3	State specific objectives, including any prespecified hypotheses	4
**Methods**			
Study design	4	Present key elements of study design early in the paper	5
Setting	5	Describe the setting, locations, and relevant dates, including periods of recruitment, exposure, follow-up, and data collection	5-6
Participants	6	(*a*) Give the eligibility criteria, and the sources and methods of selection of participants. Describe methods of follow-up	5,7
		(*b*) For matched studies, give matching criteria and number of exposed and unexposed	NA
Variables	7	Clearly define all outcomes, exposures, predictors, potential confounders, and effect modifiers. Give diagnostic criteria, if applicable	7
Data sources/ measurement	8*	For each variable of interest, give sources of data and details of methods of assessment (measurement). Describe comparability of assessment methods if there is more than one group	5-6
Bias	9	Describe any efforts to address potential sources of bias	7
Study size	10	Explain how the study size was arrived at	5
Quantitative variables	11	Explain how quantitative variables were handled in the analyses. If applicable, describe which groupings were chosen and why	7
Statistical methods	12	(*a*) Describe all statistical methods, including those used to control for confounding	7
		(*b*) Describe any methods used to examine subgroups and interactions	7
		(*c*) Explain how missing data were addressed	NA
		(*d*) If applicable, explain how loss to follow-up was addressed	NA
		(*e*) Describe any sensitivity analyses	7
**Results**			
Participants	13*	(a) Report numbers of individuals at each stage of study—eg numbers potentially eligible, examined for eligibility, confirmed eligible, included in the study, completing follow-up, and analysed	5
		(b) Give reasons for non-participation at each stage	5
		(c) Consider use of a flow diagram	5
Descriptive data	14*	(a) Give characteristics of study participants (eg demographic, clinical, social) and information on exposures and potential confounders	8
		(b) Indicate number of participants with missing data for each variable of interest	5
		(c) Summarise follow-up time (eg, average and total amount)	NA
Outcome data	15*	Report numbers of outcome events or summary measures over time	8

For peer review only - http://bmjopen.bmj.com/site/about/guidelines.xhtml

Main results	16	(*a*) Give unadjusted estimates and, if applicable, confounder-adjusted estimates and their precision (eg, 95% confidence interval). Make clear which confounders were adjusted for and why they were included	9-10
		(*b*) Report category boundaries when continuous variables were categorized	9-10
		(*c*) If relevant, consider translating estimates of relative risk into absolute risk for a meaningful time period	NA
Other analyses	17	Report other analyses done—eg analyses of subgroups and interactions, and sensitivity analyses	9
**Discussion**			
Key results	18	Summarise key results with reference to study objectives	10
Limitations	19	Discuss limitations of the study, taking into account sources of potential bias or imprecision. Discuss both direction and magnitude of any potential bias	12
Interpretation	20	Give a cautious overall interpretation of results considering objectives, limitations, multiplicity of analyses, results from similar studies, and other relevant evidence	10-11
Generalisability	21	Discuss the generalisability (external validity) of the study results	11
**Other information**			
Funding	22	Give the source of funding and the role of the funders for the present study and, if applicable, for the original study on which the present article is based	13

*Give information separately for exposed and unexposed groups.

Note: An Explanation and Elaboration article discusses each checklist item and gives methodological background and published examples of transparent reporting. The STROBE checklist is best used in conjunction with this article (freely available on the Web sites of PLoS Medicine at http://www.plosmedicine.org/, Annals of Internal Medicine at http://www.annals.org/, and Epidemiology at http://www.epidem.com/). Information on the STROBE Initiative is available at http://www.strobe-statement.org.

For peer review only

For peer review only - http://bmjopen.bmj.com/site/about/guidelines.xhtml

BMJ Open

Association between non-HDLC and 1-year prognosis in patients with spontaneous intracerebral hemorrhage: a prospective cohort study from 13 hospitals in Beijing

Journal:	*BMJ Open*
Manuscript ID	bmjopen-2022-061241.R1
Article Type:	Original research
Date Submitted by the Author:	15-Jul-2022
Complete List of Authors:	Wang, Yu; Beijing Tiantan Hospital,<br>Wu, Jianwei; Beijing Tiantan Hospital<br>Wang, Anxin; Beijing Tiantan Hospital<br>Jiang, Ruixuan; Beijing Tiantan Hospital, Department of neurology<br>Wang, Wenjuan; Beijing Tiantan Hospital, Department of Neurology<br>Zhao, Xingquan; Beijing Tiantan Hospital, Neurology
**Primary Subject Heading**:	Neurology
Secondary Subject Heading:	Medical management
Keywords:	Stroke < NEUROLOGY, Lipid disorders < DIABETES & ENDOCRINOLOGY, Risk management < HEALTH SERVICES ADMINISTRATION & MANAGEMENT

SCHOLARONE™
Manuscripts

I, the Submitting Author has the right to grant and does grant on behalf of all authors of the Work (as defined in the below author licence), an exclusive licence and/or a non-exclusive licence for contributions from authors who are: i) UK Crown employees; ii) where BMJ has agreed a CC-BY licence shall apply, and/or iii) in accordance with the terms applicable for US Federal Government officers or employees acting as part of their official duties; on a worldwide, perpetual, irrevocable, royalty-free basis to BMJ Publishing Group Ltd ("BMJ") its licensees and where the relevant Journal is co-owned by BMJ to the co-owners of the Journal, to publish the Work in this journal and any other BMJ products and to exploit all rights, as set out in our licence.

The Submitting Author accepts and understands that any supply made under these terms is made by BMJ to the Submitting Author unless you are acting as an employee on behalf of your employer or a postgraduate student of an affiliated institution which is paying any applicable article publishing charge ("APC") for Open Access articles. Where the Submitting Author wishes to make the Work available on an Open Access basis (and intends to pay the relevant APC), the terms of reuse of such Open Access shall be governed by a Creative Commons licence – details of these licences and which Creative Commons licence will apply to this Work are set out in our licence referred to above.

Other than as permitted in any relevant BMJ Author's Self Archiving Policies, I confirm this Work has not been accepted for publication elsewhere, is not being considered for publication elsewhere and does not duplicate material already published. I confirm all authors consent to publication of this Work and authorise the granting of this licence.

For peer review only

Association between non-HDLC and 1-year prognosis in patients with spontaneous intracerebral hemorrhage: a prospective cohort study from 13 hospitals in Beijing

Yu Wang,[1,2,3*] Jianwei Wu,[1,2*] Anxin Wang,[1,2] Ruixuan Jiang,[1,2] Wenjuan Wang,[1,2#] Xingquan Zhao[1,2,4#]

[1] Department of Neurology, Beijing Tiantan Hospital, Capital Medical University, Beijing, China

[2] China National Clinical Research Center for Neurological Diseases, Beijing, China

[3] Department of Neurology, Beijing Hospital, National Center of Gerontology, Beijing, China

[4] Research Unit of Artificial Intelligence in Cerebrovascular Disease, Chinese Academy of Medical Sciences, Beijing, China

*YW and JW contributed equally and share the first authorship.

The authors declare that they have no conflicts of interest.

#Correspondence to: Wenjuan Wang and Xingquan Zhao

Address: No. 119 South 4th Ring West Road, Fengtai District, Beijing 100070, China.

Telephone: 086-10-59975549   Fax: 086-10-59975549

Email: tong_ttyy@163.com (Orcid ID: 0000-0002-2572-2718) or zxq@vip.163.com (Orcid ID: 0000-0001-8345-5147)

Keywords: non-HDLC, intracerebral hemorrhage, prognosis, risk factors, mRS

Total number of tables and figures: 2 tables and 2 figures.

Word Count: 3573

For peer review only - http://bmjopen.bmj.com/site/about/guidelines.xhtml

ABSTRACT

Objectives: Previous studies suggested an inverse association between lipoprotein cholesterols and bleeding risk, while limited data was available about the predictive value of lipoproteins on intracerebral hemorrhage (ICH). Our recent research series showed that higher non-high-density lipoprotein cholesterol (non-HDLC) was an independent predictor of favourable 3-month outcome in ICH patients, we thus aimed to further investigate the association between non-HDLC levels and 1-year functional outcomes after ICH.

Design: Prospective multicenter cohort study.

Setting: 13 hospitals in Beijing, China.

Participants: A total of 666 ICH patients were included between December 2014 and September 2016.

Methods: Non-HDLC was calculated by subtracting HDL-C from TC**.** Patients were then grouped by non-HDLC levels into three categories: <3.4mmol/L, 3.4-4.2mmol/L, and ≥4.2mmol/L. Both the univariate and multivariate logistic regressions were used to assess the association between non-HDLC levels and 1-year unfavorable functional outcomes (modified Rankin Scale ≥3) in ICH patients. Moreover, sensitivity analysis was performed in ICH patients without statin use after admission.

Results: There were 33.5% (223/666) ICH patients identified with unfavorable functional outcomes at 1-year follow-up. In the univariate analysis, patients who achieved non-HDLC levels above 4.2 mmol/L had a 49% decreased risk of 1-year poor prognosis (OR 0.51, 95% CI 0.33-0.81). However, non-HDLC did not retain its independent prognostic value in multivariate analysis, the fully adjusted OR values were 1.00 (reference), 1.06 (0.63, 1.79), and 0.83 (0.45, 1.54) from the lowest to the highest non-HDLC group. Moreover, statin use after ICH onset made no difference to the long-term prognosis.

Conclusions: Non-HDLC was not an independent predictor for 1-year functional outcome in ICH patients,

For peer review only - http://bmjopen.bmj.com/site/about/guidelines.xhtml

For peer review only

irrespective of post-stroke statin use. The predictive value of well-recognized confounding factors was more dominant than non-HDLC on long-term prognosis.

Strengths and limitations of this study

- A multicenter, prospective, cohort study included 666 ICH patients from a total of 13 hospitals in Beijing.
- Our study filled the vacancy about the association between non-HDLC and 1-year functional outcomes, simultaneously shed light on the diverse impacts of non-HDLC on short-term and long-term prognosis in ICH patients.
- Sensitivity analysis was performed to evaluate the association between non-HDLC and 1-year functional outcomes in ICH patients with post-stroke statin use.
- Data regarding radiological information and antithrombotic treatment were unavailable, further exploration is needed to verify our results.

For peer review only

For peer review only - http://bmjopen.bmj.com/site/about/guidelines.xhtml

INTRODUCTION

Intracerebral hemorrhage (ICH) is the second most common subtype of stroke, leading to severe disability and mortality.[1] Based on the nationally representative stroke survey in China published recently, ICH accounts for 25% of all strokes with an overall age-standardized incidence of 66.2 per 100,000 person-years.[2] Despite rapid advances in medicine, the management of ICH remains supportive without significant breakthroughs.[3] Approximately 30-48% of ICH patients died within one month in low- to middle-income countries and only 12-39% of survivors could achieve long-term functional independence.[1, 4]

The conventional view on lipid-lowering targets goes “the lower, the better” in patients with atherosclerotic cardiovascular disease. However, previous epidemiology studies suggested an inverse association between lipoprotein cholesterols and ICH risk, hematoma expansion, and mortality.[5, 6] Much remains to be discussed on the predictive value of lipoproteins on ICH. Our recent research series showed that low serum lipid levels were independent predictors of 3-month poor prognosis in ICH patients, and non-high-density lipoprotein cholesterol (non-HDLC) was the optimal parameter with high specificity.[7, 8] However, the literature has scant information regarding the association between non-HDLC and long-term ICH prognosis.

We thus aimed to investigate the association between serum non-HDLC levels and 1-year functional outcomes after ICH in this prospective cohort study.

METHODS

Study population

The study was conducted in accordance with the Declaration of Helsinki and was approved by

For peer review only - http://bmjopen.bmj.com/site/about/guidelines.xhtml

the Institutional Review Board of the Beijing Tiantan Hospital (KY2014-023-02). All participants or their legal representatives provided written informed consent.

Our study is a multicenter, prospective, cohort study conducted in a total of 13 hospitals, evaluating the medical quality of cerebral hemorrhage on different etiologies in Beijing. From December 2014 to September 2016, 1964 consecutive ICH patients agreed to participate in the study. A total of 1881 patients met the following inclusion criteria: (1) aged 18 years or older, (2) had their first CT scan done within 72h after symptom onset. After excluding 159 secondary ICH patients (caused by trauma, tumor, aneurysm, arteriovenous malformation, coagulopathy, or other causes) and 20 patients diagnosed as primary ventricular hemorrhage, 1702 patients with primary intraparenchymal hemorrhage were included. Moreover, 294 patients underwent surgical procedures (including craniotomy hematoma removal, hematoma puncture, extraventricular drainage, and so on), 15 patients with anticoagulant therapy before symptom onset, 588 patients with missing data on the non-HDLC level, and 139 patients lost to follow-up at 1-year were excluded. Eventually, 666 patients with spontaneous ICH from 13 sites were included (Figure 1).

Baseline information

Demographic information including age, sex, onset to admission time, past medical history (including hypertension, diabetes mellitus, hyperlipidemia, cerebral infarction, and ICH), personal habits (including smoking and drinking status), and medication history (including antiplatelet and statin therapy) of each patient was collected using a standard questionnaire at baseline. Neurological deficits were assessed using the National Institute of Health Stroke Scale (NIHSS)

For peer review only - http://bmjopen.bmj.com/site/about/guidelines.xhtml

and Glasgow Coma Scale (GCS) score by experienced neurologists on admission. Meanwhile, systolic and diastolic blood pressure (BP) were measured. A cranial CT scan was performed on admission and hematoma volume was then calculated as ABC/2 volumetric formula at each site.[9] The location of hematoma was further subdivided into supratentorial and infratentorial regions. ICH score was calculated based on five parameters, GCS score, ICH volume, the presence of intraventricular extension, location of hematoma, and age.[10]

For peer review only

Measurement of non-HDLC levels and other biochemical parameters

Blood samples were drawn from the antecubital vein the next morning after an overnight fast and analyzed within 4h. Total cholesterol (TC) was measured using the end-point test method and high-density lipoprotein cholesterol (HDL-C) was measured using a direct method. Non-HDLC was thus calculated by subtracting HDL-C from TC. Based on the National Lipid Association Recommendations,[11] non-HDLC levels were categorized into five groups: desirable, <3.4mmol/L; above desirable, 3.4-4.2mmol/L; borderline high, 4.2-5.0mmol/L; high, 5.0-5.8 mmol/L; and very high, ≥5.8 mmol/L. Accordingly, we integrated the last three groups into one group (≥4.2mmol/L) due to the limited number of patients.

For other biochemical parameters, random blood glucose was measured via the hexokinase/glucose-6-phosphate dehydrogenase method, serum creatinine was measured through rate reflectance spectrophotometry, white blood cell (WBC) together with platelet count were performed on EDTA with an ADVIA 120 counter (Siemens Healthcare Diagnostics, Saint-Denis, France).

For peer review only - http://bmjopen.bmj.com/site/about/guidelines.xhtml

Follow-up information and definition of 1-year ICH prognosis

Patients were followed up at 1-year after ICH onset via telephone interviews. Follow-up evaluation was performed by neurologists who were blinded to prognostic factors. 1-year prognosis of patients was evaluated by modified Ranking Scale (mRS) score and categorized as favorable (mRS<3) and unfavorable functional outcome groups (mRS≥3).

Patient and public involvement

No patients were involved.

Statistical analysis

The patients were divided into three groups according to the clinical diagnosis of abnormal non-HDLC levels. Continuous variables were presented as median with interquartile range (IQR), categorical variables were described as count with percentage. The group differences of continuous variables were analyzed using ANOVA or Kruskal-Wallis test as appropriate, and for categorical variables, chi-squared tests were performed. Logistic regression was used to evaluate the association between non-HDLC levels and 1-year prognosis of ICH patients, with the lowest non-HDLC group (<3.4mmol/L) used as the reference. Both the univariate and multivariate analyses were conducted to estimate the odds ratios (ORs) and 95% confidence intervals (CIs). Multiple regression models were run as follows. Model 1 was adjusted for age and sex. Model 2 was adjusted for variates in model 1 plus premorbid mRS score (<3 or ≥3), history of ICH, glucose on admission, WBC on admission, baseline hematoma volume, hematoma location, time from onset to initial non-contrast CT, GCS score at admission, and systolic BP. *P*-values for trend were

For peer review only - http://bmjopen.bmj.com/site/about/guidelines.xhtml

conducted using the three categories of non-HDLC as ordinal variables in the model. Additionally, sensitivity analysis was performed in ICH patients without statin use after admission (n=589). A 2-sided value of $p<0.05$ was considered statistically significant. All statistical analyses were performed using SAS software, version 9.4 (SAS Institute, Cary, NC, USA).

RESULTS

A total of 666 eligible patients were included, with a mean age of 59 years old (ranging from 51 to 68) and 69.1% (460/666) of them were males. Amongst them, 33.5% (223/666) were identified as 1-year poor outcomes, the proportion of which were 38.4%, 30.3%, and 24.2% from <3.4mmol/L group to ≥4.2mmol/L group.

Baseline characteristics

There were significant differences in age, prior statin use, diastolic BP, glucose on admission, WBC on admission, and statin use after admission among the three categories of non-HDLC levels ($p<0.05$, Table 1). Those with higher lipid levels were more likely to be younger, not a prior statin user, having higher diastolic BP and glucose on admission. While no statistical significance was observed in sex, premorbid mRS scale, onset to admission time, past medical history, personal habits, prior antiplatelet use, NIHSS score, GCS score, SBP, creatinine, infections, time from onset to initial NCCT, hematoma volume, hematoma location, and ICH score between the three groups.

Table 1. Baseline characteristics of participants according to non-HDLC levels.

For peer review only - http://bmjopen.bmj.com/site/about/guidelines.xhtml

	Total	non-HDLC levels			P-value
		<3.4mmol/L	3.4-4.2mmol/L	≥4.2mmol/L	
n (%)	666	359 (53.9)	175 (26.3)	132 (19.8)	
Age, years	59 (51, 68)	61 (53, 70)	57 (49, 67)	54 (48, 64)	<0.001
Male, n (%)	460 (69.1)	258 (71.9)	120 (68.6)	82 (62.1)	0.116
Onset to admission time, h	4.0 (1.8, 11.9)	3.8 (1.7, 11.1)	4.0 (2.0, 11.0)	4.0 (1.8, 14.7)	0.840
Premorbid mRS score					0.614
mRS<3	643 (96.5)	345 (96.1)	171 (97.7)	127 (96.2)	
mRS≥3	23 (3.5)	14 (3.9)	4 (2.3)	5 (3.8)	
Hypertension, n (%)	479 (71.9)	256 (71.3)	124 (70.9)	99 (75.0)	0.676
Diabetes mellitus, n (%)	106 (15.9)	55 (15.3)	29 (16.6)	22 (16.7)	0.902
Hyperlipidemia, n (%)	68 (10.2)	36 (10.0)	18 (10.3)	14 (10.6)	0.982
History of CI, n (%)	102 (15.3)	58 (16.2)	27 (15.4)	17 (12.9)	0.670
History of ICH, n (%)	20 (3.0)	15 (4.2)	3 (1.7)	2 (1.5)	0.141
Smoking, n (%)	223 (33.5)	127 (35.4)	57 (32.6)	39 (29.6)	0.458
Drinking, n (%)	256 (38.4)	139 (38.7)	69 (39.4)	48 (36.4)	0.850
Prior antiplatelet use, n (%)	110 (16.5)	61 (17.0)	28 (16.0)	21 (15.9)	0.771
Prior statin use, n (%)	44 (6.6)	31 (8.6)	10 (5.7)	3 (2.3)	0.036
NIHSS score on admission	8 (3, 13)	9 (3, 15)	7 (3, 13)	5 (2, 12)	0.083
GCS score on admission	14 (12, 15)	14 (12, 15)	15 (13, 15)	15 (13, 15)	0.063
SBP on admission, mmHg	160 (149, 183)	160 (150, 180)	160 (145, 183)	162 (150, 183)	0.564
DBP on admission, mmHg	95 (83, 105)	92 (80, 102)	96 (85, 106)	97 (85, 109)	0.024
Glucose on admission, mmol/L	6.9 (5.9, 8.4)	6.6 (5.8, 8.1)	7.0 (5.9, 8.6)	7.1 (6.0, 9.3)	0.032
WBC on admission, 10^9/L	8.4 (6.6, 10.9)	8.1 (6.3, 10.7)	9.1 (7.0, 11.7)	7.1 (6.0, 9.3)	0.007
Platelets on admission, 10^9/L	212 (175, 252)	202 (164, 238)	218 (180, 259)	230 (192, 265)	<0.001
Creatinine on admission, µmol/L	64.0 (53.0, 77.3)	64.6 (54.0, 76.4)	65.0 (52.3, 79.0)	62.0 (50.1, 76.0)	0.223
Statin use after admission, n (%)	77 (11.6)	19 (5.3)	30 (17.1)	28 (21.2)	<0.001
Infections, n (%)	136 (20.4)	77 (21.5)	39 (22.3)	20 (15.2)	0.239
Time from onset to initial NCCT, h	5.2 (2.3, 16.7)	5.2 (2.2, 14.8)	5.1 (2.3, 19.6)	4.8 (2.3, 19.4)	0.738
Baseline hematoma volume, ml	10.5 (5.0, 23.4)	10.7 (5.0, 25.0)	10.4 (5.5, 23.1)	10.0 (4.9, 16.8)	0.379
Hematoma location					0.251
Supratentorial, n (%)	599 (89.7)	327 (91.2)	155 (88.2)	117 (87.5)	
Infratentorial, n (%)	67 (10.3)	31 (8.8)	23 (11.8)	16 (12.5)	
ICH score	0 (0, 1)	0 (0, 1)	0 (0, 1)	0 (0, 1)	0.447

Values are (%) for categorical variables and median (IQR) for continuous variables.
mRS, modified Rankin Scale; CI, cerebral infarction; ICH, intracerebral hemorrhage; NIHSS, National Institutes of Health Stroke Scale; GCS, Glasgow Coma Scale; SBP, systolic blood pressure; DBP, diastolic blood pressure; WBC, white blood cells; NCCT, non-contrast CT.

Correlation between baseline non-HDLC and 1-year prognosis in ICH patients

In the univariate analysis, higher non-HDLC levels were significantly associated with decreased risk of 1-year poor outcome (p=0.002). Patients who achieved non-HDLC above 4.2mmol/L had a 49% lower risk of poor functional outcome at 1 year (OR 0.51, 95% CI 0.33-0.81). While no statistical difference was retained after adjusting for age, sex, and potential confounding factors

For peer review only - http://bmjopen.bmj.com/site/about/guidelines.xhtml

(p>0.05). In the fully adjusted model (Model 2), the OR values were 1.00 (reference), 1.06 (0.63, 1.79), and 0.83 (0.45, 1.54) from the lowest to the highest non-HDLC group. Moreover, the results maintained consistency in sensitivity analysis among patients without statin use after admission (p=0.842, Table 2).

Table 2. Odds ratios and 95% CI for 1-year poor outcome (mRS ≥3) according to non-HDLC levels.

	non-HDLC levels			Continuous scale	*P* for trend
	<3.4mmol/L	3.4-4.2mmol/L	⩾4.2mmol/L		
1-year poor outcome, n (%)	138 (38.4)	53 (30.3)	32 (24.2)		
Univariate analysis	Ref.	0.70 (0.47, 1.02)	0.51 (0.33, 0.81)	0.71 (0.58, 0.88)	0.002
Multivariate analysis					
Model 1	Ref.	0.82 (0.54, 1.23)	0.66 (0.41, 1.06)	0.81 (0.65, 1.02)	0.075
Model 2	Ref.	1.06 (0.63, 1.79)	0.83 (0.45, 1.54)	0.89 (0.76, 1.05)	0.694
Sensitivity analysis	Ref.	0.92 (0.53, 1.61)	1.12 (0.58, 2.16)	0.92 (0.78, 1.08)	0.842

Data are OR (95% CI) unless otherwise stated.
Model 1 adjusted for age and sex.
Model 2 adjusted for variates in model 1 plus premorbid mRS score (<3 or ≥3), history of ICH, glucose on admission, WBC on admission, baseline hematoma volume, hematoma location, time from onset to initial non-contrast CT, GCS score at admission, systolic blood pressure.
Sensitivity analysis was performed in ICH patients without statin use after admission (n=589), and adjusted for variates in model 2.

Notably, age, premorbid mRS score (<3 or ≥3), and baseline hematoma volume were positively associated with 1-year poor prognosis in the multivariate analysis. Whereas, higher GCS score at admission was an independent predictor of favorable outcomes. Additional detailed information was given in Figure 2.

DISCUSSION

This study provided evidence on the association between non-HDLC levels and long-term functional outcomes in ICH patients. Although non-HDLC was a significant 1-year predictor in

For peer review only - http://bmjopen.bmj.com/site/about/guidelines.xhtml

univariate analysis, it did not retain its independent prognostic value in multivariate analysis. Moreover, statin use after ICH onset made no difference to the long-term prognosis.

In our study, the prevalence of 1-year functional independence in ICH patients was 66.5% (443/666), far outweighing the data previously reported.[4] According to the inclusion and exclusion criteria, severe cases who underwent surgical treatment or lost to follow-up were not enrolled. It is noteworthy that per 1 mmol/L increment in non-HDLC yielded a 29% decreased risk of 1-year poor prognosis (crude OR 0.71, 95% CI 0.58-0.88). However, contrary to our previous research finding of the independent role of non-HDLC on short-term functional outcomes,[7] the results of this study showed that age, premorbid mRS score, baseline hematoma volume, admission GCS score, rather than non-HDLC level, were independent predictors for long-term functional outcomes in ICH patients. The validated predictors mentioned above kept high conformity with the items in ICH Functional Outcome Score, an effective prognostic model for 1-year poor functional outcomes after ICH,[12] whereas the absolute magnitude effect of low non-HDLC level on ICH prognosis was likely to be small and overshadowed with time. Beyond that, the amount of rehabilitation with functional gains might also related.[13]

It was reported that low levels of LDL-C and TC were associated with hematoma expansion.[14, 15] As containing all the atherogenic lipoproteins, non-HDLC was served as the preferred target of lipid-lowering therapy.[16] The potential mechanisms regarding the association between hypolipidemia and hematoma expansion, including impaired endothelial integrity,[17] necrotic medial smooth muscle cells,[18] increased erythrocyte fragility,[19] inhibited platelet aggregation,[20] and the resultant incident cerebral microbleeds.[21] Despite the theoretical basis, our study failed to show an independent correlation between non-HDLC levels and 1-year

For peer review only - http://bmjopen.bmj.com/site/about/guidelines.xhtml

functional outcomes in ICH patients. The secondary injury caused by low levels of lipoproteins in ICH patients was associated with short-term prognosis (30-day, 3-month),[22, 23] while its impact on long-term prognosis (1-year) was negative, which merits further investigation due to the limited sample size and incomplete neuroimaging data on hematoma expansion in our study.

Statin treatment is another major concern,[24] there were respectively 6.6% (44/666) and 11.6% (77/666) patients with pre- and post-stroke statin use in our study. Two recent meta-analyses concluded that there was no evidence to suggest pre-stroke statin therapy may increase bleeding risk in the context of ICH.[25, 26] Whether to start, continue, or stop statin treatment in ICH patients has aroused great concern, we thus conducted a sensitivity analysis to evaluate the effect of statin exposure after admission on ICH prognosis. No significant difference was detected between non-HDLC levels and 1-year prognosis in ICH patients in our study, irrespective of post-stroke statin use. A recent review indicated that statin should be applied after weighing the pros and cons given its pleiotropic as well as lipid-lowering effects.[27] Because of the relatively low stain exposure rate in our study, it is necessary to conduct randomized controlled trials around this topic.

Our study filled the vacancy about the association between non-HDLC and 1-year functional outcomes, simultaneously shed light on the diverse impacts of non-HDLC on short-term and long-term prognosis in ICH patients. Nonetheless, there are still some limitations. First, the follow-up radiological information was unavailable, which makes it difficult to verify the intermediate role of hematoma expansion between non-HDLC and poor prognosis. Secondly, ICH caused by cerebral amyloid angiopathy has a higher rebleeding risk than hypertensive one,[28] while data regarding the cause of ICH was not documented in our study. Despite no correlation

For peer review only - http://bmjopen.bmj.com/site/about/guidelines.xhtml

was observed between the history of ICH and 1-year functional outcome, the impact of ICH etiology merits further investigation. Thirdly, medication therapy regarding antiplatelet or anticoagulation agents were not included in the multivariate analysis, whereas accumulating researches proved that antithrombotic treatment increased the risk of cerebral microbleeds as well as future ICH.[29, 30] Although we collected pre-ictus antiplatelet use, restricted by the small sample size, further research is needed to provide insight into the relationship. Moreover, since our study based on a highly selected population with small hematoma and relatively good neurologic status to achieve precise research, the findings cannot be generalized to the whole ICH population.

CONCLUSION

In conclusion, non-HDLC was not an independent predictor for 1-year functional outcome in ICH patients, irrespective of post-stroke statin use. The predictive value of well-recognized confounding factors was more dominant than non-HDLC on long-term poor prognosis. Further prospective studies are needed to assess the impact of lower non-HLDC levels on ICH prognosis.

Author affiliations

1 China National Clinical Research Center for Neurological Diseases, Beijing, China

2 Department of Neurology, Beijing Tiantan Hospital, Capital Medical University, Beijing, China

3 Research Unit of Artificial Intelligence in Cerebrovascular Disease, Chinese Academy of Medical Sciences, Beijing, China

For peer review only - http://bmjopen.bmj.com/site/about/guidelines.xhtml

Contributors YW and JW performed the experiments, interpreted the results of statistical analysis, and drafted the manuscript. AW conducted the statistical analysis and interpreted the data. RJ revised the manuscript for intellectual content. WW and XZ had full access to all of the data and take responsibility for the integrity of the data and the accuracy of the data analysis.

Funding Our study was supported by grants from the Chinese Academy of Medical Sciences Innovation Fund for Medical Sciences (2019-I2M-5-029), Beijing Natural Science Foundation (Z200016), Beijing Municipal Committee of Science and Technology (Z201100005620010), and Ministry of Science and Technology of the People’s Republic of China (2018YFC1705003).

Competing interests None declared.

Patient consent for publication Not applicable.

Ethics approval The study was approved by the Central Institutional Review Board of Beijing Tiantan Hospital (KY2014-023-02) and written informed consent was obtained.

Provenance and peer review Not commissioned; externally peer reviewed.

Data sharing statement Some or all datasets generated during and/or analyzed during the current study are not publicly available but are available from the corresponding author on reasonable request.

References

1. An SJ, Kim TJ, Yoon BW. Epidemiology, Risk Factors, and Clinical Features of Intracerebral Hemorrhage: An Update. J Stroke 2017;19:3-10.
2. Wang W, Jiang B, Sun H, et al. Prevalence, Incidence, and Mortality of Stroke in China: Results from a Nationwide Population-Based Survey of 480 687 Adults. Circulation 2017;135:759-771.

For peer review only - http://bmjopen.bmj.com/site/about/guidelines.xhtml

3. Hemphill JC, 3rd, Greenberg SM, Anderson CS, et al. Guidelines for the Management of Spontaneous Intracerebral Hemorrhage: A Guideline for Healthcare Professionals From the American Heart Association/American Stroke Association. Stroke 2015;46:2032-2060.
4. Feigin VL, Lawes CM, Bennett DA, Barker-Collo SL, Parag V. Worldwide stroke incidence and early case fatality reported in 56 population-based studies: a systematic review. Lancet Neurol 2009;8:355-369.
5. Wang X, Dong Y, Qi X, Huang C, Hou L. Cholesterol levels and risk of hemorrhagic stroke: a systematic review and meta-analysis. Stroke 2013;44:1833-1839.
6. Chang JJ, Katsanos AH, Khorchid Y, et al. Higher low-density lipoprotein cholesterol levels are associated with decreased mortality in patients with intracerebral hemorrhage. Atherosclerosis 2018;269:14-20.
7. Feng H, Wang X, Wang W, Zhao X. Association Between Non-high-density Lipoprotein Cholesterol and 3-Month Prognosis in Patients With Spontaneous Intracerebral Hemorrhage. Front Neurol 2020;11:920.
8. Feng H, Wang X, Wang W, Zhao X. Lipid Levels and 3-Month Prognosis After Spontaneous Intracerebral Hemorrhage in Women. Front Neurol 2021;12:690194.
9. Kothari RU, Brott T, Broderick JP, et al. The ABCs of measuring intracerebral hemorrhage volumes. Stroke 1996;27:1304-1305.
10. Hemphill JC, 3rd, Bonovich DC, Besmertis L, Manley GT, Johnston SC. The ICH score: a simple, reliable grading scale for intracerebral hemorrhage. Stroke 2001;32:891-897.
11. Jacobson TA, Ito MK, Maki KC, et al. National lipid association recommendations for patient-centered management of dyslipidemia: part 1--full report. J Clin Lipidol 2015;9:129-169.
12. Ji R, Shen H, Pan Y, et al. A novel risk score to predict 1-year functional outcome after intracerebral hemorrhage and comparison with existing scores. Crit Care 2013;17:R275.
13. Kamo T, Momosaki R, Suzuki K, et al. Effectiveness of Intensive Rehabilitation Therapy on Functional Outcomes After Stroke: A Propensity Score Analysis Based on Japan Rehabilitation Database. J Stroke Cerebrovasc Dis 2019;28:2537-2542.
14. Rodriguez-Luna D, Rubiera M, Ribo M, et al. Serum low-density lipoprotein cholesterol level predicts hematoma growth and clinical outcome after acute intracerebral hemorrhage. Stroke 2011;42:2447-2452.
15. Broderick JP, Diringer MN, Hill MD, et al. Determinants of intracerebral hemorrhage growth: an exploratory analysis. Stroke 2007;38:1072-1075.
16. National Cholesterol Education Program Coordinating Committee. Third Report of the National Cholesterol Education Program (NCEP) Expert Panel on Detection, Evaluation, and Treatment of High Blood Cholesterol in Adults (Adult Treatment Panel III) final report. Circulation 2002;106:3143-3421.
17. Bang OY, Saver JL, Liebeskind DS, et al. Cholesterol level and symptomatic hemorrhagic transformation after ischemic stroke thrombolysis. Neurology 2007;68:737-742.
18. Ooneda G, Yoshida Y, Suzuki K, et al. Smooth muscle cells in the development of plasmatic arterionecrosis, arteriosclerosis, and arterial contraction. Blood Vessels 1978;15:148-156.
19. Yamori Y, Nara Y, Horie R, Ooshima A. Abnormal membrane characteristics of erythrocytes in rat models and men with predisposition to stroke. Clin Exp Hypertens 1980;2:1009-1021.
20. Chui DH, Marotta F, Rao ML, Liu DS, Zhang SC, Ideo C. Cholesterol-rich LDL perfused at physiological LDL-cholesterol concentration induces platelet aggregation and PAF-

For peer review only - http://bmjopen.bmj.com/site/about/guidelines.xhtml

acetylhydrolase activation. Biomed Pharmacother 1991;45:37-42.

21. Lee SH, Bae HJ, Yoon BW, Kim H, Kim DE, Roh JK. Low concentration of serum total cholesterol is associated with multifocal signal loss lesions on gradient-echo magnetic resonance imaging: analysis of risk factors for multifocal signal loss lesions. Stroke 2002;33:2845-2849.
22. Rodriguez-Luna D, Rubiera M, Ribo M, et al. Ultraearly hematoma growth predicts poor outcome after acute intracerebral hemorrhage. Neurology 2011;77:1599-1604.
23. Sembolini A, Romoli M, Pannacci U, et al. Acute hematoma expansion after spontaneous intracerebral hemorrhage: risk factors and impact on long-term prognosis. Neurol Sci 2020;41:2503-2509.
24. Goldstein LB, Amarenco P, Szarek M, et al. Hemorrhagic stroke in the Stroke Prevention by Aggressive Reduction in Cholesterol Levels study. Neurology 2008;70:2364-2370.
25. Hackam DG, Woodward M, Newby LK, et al. Statins and intracerebral hemorrhage: collaborative systematic review and meta-analysis. Circulation 2011;124:2233-2242.
26. Ziff OJ, Banerjee G, Ambler G, Werring DJ. Statins and the risk of intracerebral haemorrhage in patients with stroke: systematic review and meta-analysis. J Neurol Neurosurg Psychiatry 2019;90:75-83.
27. Endres M, Nolte CH, Scheitz JF. Statin Treatment in Patients With Intracerebral Hemorrhage. Stroke 2018;49:240-246.
28. Pasi M, Charidimou A, Boulouis G, et al. Mixed-location cerebral hemorrhage/microbleeds: Underlying microangiopathy and recurrence risk. Neurology 2018;90:e119-e126.
29. Qiu J, Ye H, Wang J, Yan J, Wang J, Wang Y. Antiplatelet Therapy, Cerebral Microbleeds, and Intracerebral Hemorrhage: A Meta-Analysis. Stroke 2018;49:1751-1754.
30. Lioutas VA, Goyal N, Katsanos AH, et al. Optimization of risk stratification for anticoagulation-associated intracerebral hemorrhage: net risk estimation. J Neurol 2020;267:1053-1062.

For peer review only

For peer review only - http://bmjopen.bmj.com/site/about/guidelines.xhtml

Figure Legends

Figure 1. Flow chart for selection of study participants.

ICH, intracerebral hemorrhage; non-HDLC, non-high-density lipoprotein cholesterol.

Figure 2. Multivariate predictors of 1-year poor outcome among ICH patients.

Non-HDLC, non-high-density lipoprotein cholesterol; ICH, intracerebral hemorrhage; mRS, modified Rankin Scale; WBC, white blood cells; NCCT, non-contrast CT; GCS, Glasgow Coma Scale; SBP, systolic blood pressure.

For peer review only

For peer review only - http://bmjopen.bmj.com/site/about/guidelines.xhtml

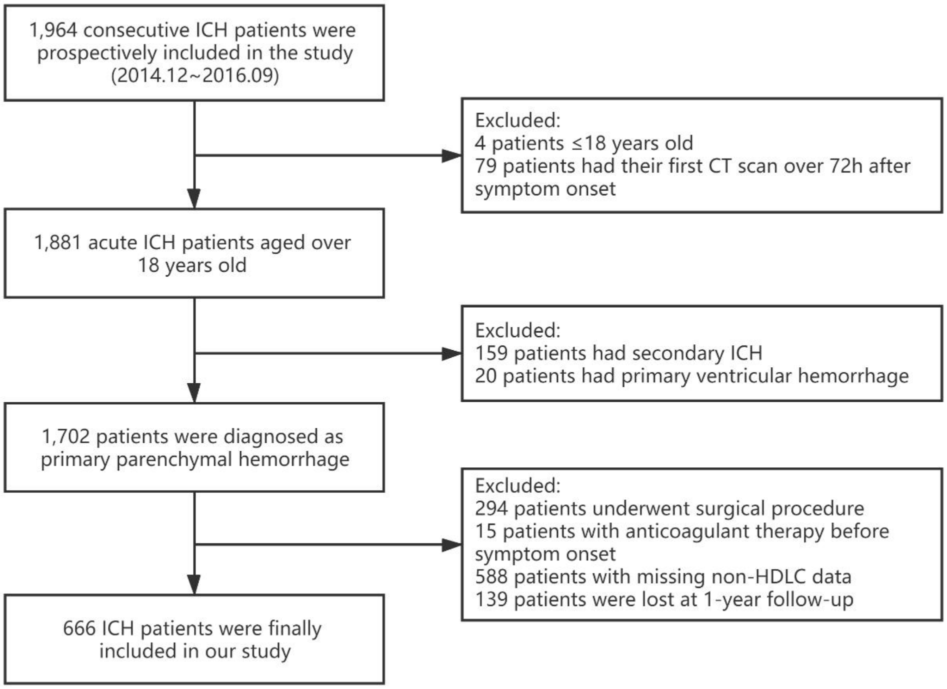

Figure 1. Flow chart for selection of study participants.
ICH, intracerebral hemorrhage; non-HDLC, non-high-density lipoprotein cholesterol.

146x108mm (220 x 220 DPI)

Variables	Poor outcome (mRS ≥3, n=178)	Odds ratio (95% CI)
non-HDLC (vs <3.4mmol/L)		
3.4-4.2mmol/L	1.06 (0.63, 1.79)	
≥4.2mmol/L	0.83 (0.45, 1.54)	
Age, years	1.08 (1.06, 1.10)	
Male	0.84 (0.52, 1.36)	
Premorbid mRS score ≥3	3.89 (1.43, 6.77)	
History of ICH	2.80 (0.95, 5.27)	
Glucose on admission, mmol/L	1.02 (0.95, 1.11)	
WBC on admission, 10E9/L	1.00 (0.93, 1.08)	
Platelet on admission, 10E9/L	1.00 (0.99, 1.00)	
Baseline hematoma volume, mL	1.02 (1.01, 1.04)	
Infratentorial hemorrhage	0.82 (0.37, 1.82)	
Tme from onset to initial NCCT, hours	0.99 (0.98, 1.01)	
GCS score on admission	0.73 (0.66, 0.80)	
SBP, mmHg	0.99 (0.99, 1.00)	

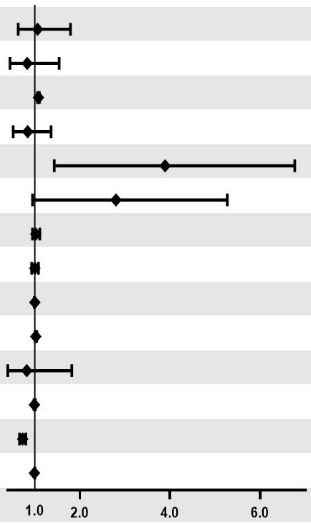

Figure 2. Multivariate predictors of 1-year poor outcome among ICH patients.
Non-HDLC, non-high-density lipoprotein cholesterol; ICH, intracerebral hemorrhage; mRS, modified Rankin Scale; WBC, white blood cells; NCCT, non-contrast CT; GCS, Glasgow Coma Scale; SBP, systolic blood pressure.

184x114mm (192 x 192 DPI)

STROBE Statement—Checklist of items that should be included in reports of ***cohort studies***

	Item No	Recommendation	Page No
**Title and abstract**	1	(*a*) Indicate the study's design with a commonly used term in the title or the abstract	1
		(*b*) Provide in the abstract an informative and balanced summary of what was done and what was found	2
**Introduction**			
Background/rationale	2	Explain the scientific background and rationale for the investigation being reported	4
Objectives	3	State specific objectives, including any prespecified hypotheses	4
**Methods**			
Study design	4	Present key elements of study design early in the paper	5
Setting	5	Describe the setting, locations, and relevant dates, including periods of recruitment, exposure, follow-up, and data collection	5-6
Participants	6	(*a*) Give the eligibility criteria, and the sources and methods of selection of participants. Describe methods of follow-up	5,7
		(*b*) For matched studies, give matching criteria and number of exposed and unexposed	NA
Variables	7	Clearly define all outcomes, exposures, predictors, potential confounders, and effect modifiers. Give diagnostic criteria, if applicable	7
Data sources/ measurement	8*	For each variable of interest, give sources of data and details of methods of assessment (measurement). Describe comparability of assessment methods if there is more than one group	5-6
Bias	9	Describe any efforts to address potential sources of bias	7
Study size	10	Explain how the study size was arrived at	5
Quantitative variables	11	Explain how quantitative variables were handled in the analyses. If applicable, describe which groupings were chosen and why	7
Statistical methods	12	(*a*) Describe all statistical methods, including those used to control for confounding	7
		(*b*) Describe any methods used to examine subgroups and interactions	7
		(*c*) Explain how missing data were addressed	NA
		(*d*) If applicable, explain how loss to follow-up was addressed	NA
		(*e*) Describe any sensitivity analyses	7
**Results**			
Participants	13*	(a) Report numbers of individuals at each stage of study—eg numbers potentially eligible, examined for eligibility, confirmed eligible, included in the study, completing follow-up, and analysed	5
		(b) Give reasons for non-participation at each stage	5
		(c) Consider use of a flow diagram	5
Descriptive data	14*	(a) Give characteristics of study participants (eg demographic, clinical, social) and information on exposures and potential confounders	8
		(b) Indicate number of participants with missing data for each variable of interest	5
		(c) Summarise follow-up time (eg, average and total amount)	NA
Outcome data	15*	Report numbers of outcome events or summary measures over time	8

For peer review only - http://bmjopen.bmj.com/site/about/guidelines.xhtml

Main results	16	(*a*) Give unadjusted estimates and, if applicable, confounder-adjusted estimates and their precision (eg, 95% confidence interval). Make clear which confounders were adjusted for and why they were included	9-10
		(*b*) Report category boundaries when continuous variables were categorized	9-10
		(*c*) If relevant, consider translating estimates of relative risk into absolute risk for a meaningful time period	NA
Other analyses	17	Report other analyses done—eg analyses of subgroups and interactions, and sensitivity analyses	9
**Discussion**			
Key results	18	Summarise key results with reference to study objectives	10
Limitations	19	Discuss limitations of the study, taking into account sources of potential bias or imprecision. Discuss both direction and magnitude of any potential bias	12
Interpretation	20	Give a cautious overall interpretation of results considering objectives, limitations, multiplicity of analyses, results from similar studies, and other relevant evidence	10-11
Generalisability	21	Discuss the generalisability (external validity) of the study results	11
**Other information**			
Funding	22	Give the source of funding and the role of the funders for the present study and, if applicable, for the original study on which the present article is based	13

*Give information separately for exposed and unexposed groups.

Note: An Explanation and Elaboration article discusses each checklist item and gives methodological background and published examples of transparent reporting. The STROBE checklist is best used in conjunction with this article (freely available on the Web sites of PLoS Medicine at http://www.plosmedicine.org/, Annals of Internal Medicine at http://www.annals.org/, and Epidemiology at http://www.epidem.com/). Information on the STROBE Initiative is available at http://www.strobe-statement.org.

For peer review only

For peer review only - http://bmjopen.bmj.com/site/about/guidelines.xhtml

BMJ Open

Association between non-HDLC and 1-year prognosis in patients with spontaneous intracerebral hemorrhage: a prospective cohort study from 13 hospitals in Beijing

Journal:	*BMJ Open*
Manuscript ID	bmjopen-2022-061241.R2
Article Type:	Original research
Date Submitted by the Author:	06-Sep-2022
Complete List of Authors:	Wang, Yu; Beijing Tiantan Hospital,<br>Wu, Jianwei; Beijing Tiantan Hospital<br>Wang, Anxin; Beijing Tiantan Hospital<br>Jiang, Ruixuan; Beijing Tiantan Hospital, Department of neurology<br>Wang, Wenjuan; Beijing Tiantan Hospital, Department of Neurology<br>Zhao, Xingquan; Beijing Tiantan Hospital, Neurology
**Primary Subject Heading**:	Neurology
Secondary Subject Heading:	Medical management
Keywords:	Stroke < NEUROLOGY, Lipid disorders < DIABETES & ENDOCRINOLOGY, Risk management < HEALTH SERVICES ADMINISTRATION & MANAGEMENT

SCHOLARONE™
Manuscripts

I, the Submitting Author has the right to grant and does grant on behalf of all authors of the Work (as defined in the below author licence), an exclusive licence and/or a non-exclusive licence for contributions from authors who are: i) UK Crown employees; ii) where BMJ has agreed a CC-BY licence shall apply, and/or iii) in accordance with the terms applicable for US Federal Government officers or employees acting as part of their official duties; on a worldwide, perpetual, irrevocable, royalty-free basis to BMJ Publishing Group Ltd ("BMJ") its licensees and where the relevant Journal is co-owned by BMJ to the co-owners of the Journal, to publish the Work in this journal and any other BMJ products and to exploit all rights, as set out in our licence.

The Submitting Author accepts and understands that any supply made under these terms is made by BMJ to the Submitting Author unless you are acting as an employee on behalf of your employer or a postgraduate student of an affiliated institution which is paying any applicable article publishing charge ("APC") for Open Access articles. Where the Submitting Author wishes to make the Work available on an Open Access basis (and intends to pay the relevant APC), the terms of reuse of such Open Access shall be governed by a Creative Commons licence – details of these licences and which Creative Commons licence will apply to this Work are set out in our licence referred to above.

Other than as permitted in any relevant BMJ Author's Self Archiving Policies, I confirm this Work has not been accepted for publication elsewhere, is not being considered for publication elsewhere and does not duplicate material already published. I confirm all authors consent to publication of this Work and authorise the granting of this licence.

For peer review only

Association between non-HDLC and 1-year prognosis in patients with spontaneous intracerebral hemorrhage: a prospective cohort study from 13 hospitals in Beijing

Yu Wang,[1,2,3*] Jianwei Wu,[1,2*] Anxin Wang,[1,2] Ruixuan Jiang,[1,2] Wenjuan Wang,[1,2#] Xingquan Zhao[1,2,4#]

[1] Department of Neurology, Beijing Tiantan Hospital, Capital Medical University, Beijing, China

[2] China National Clinical Research Center for Neurological Diseases, Beijing, China

[3] Department of Neurology, Beijing Hospital, National Center of Gerontology, Beijing, China

[4] Research Unit of Artificial Intelligence in Cerebrovascular Disease, Chinese Academy of Medical Sciences, Beijing, China

*YW and JW contributed equally and share the first authorship.

The authors declare that they have no conflicts of interest.

#Correspondence to: Wenjuan Wang and Xingquan Zhao

Address: No. 119 South 4th Ring West Road, Fengtai District, Beijing 100070, China.

Telephone: 086-10-59975549 Fax: 086-10-59975549

Email: tong_ttyy@163.com (Orcid ID: 0000-0002-2572-2718) or zxq@vip.163.com (Orcid ID: 0000-0001-8345-5147)

Keywords: non-HDLC, intracerebral hemorrhage, prognosis, risk factors, mRS

Total number of tables and figures: 2 tables and 2 figures.

Word Count: 3956

For peer review only

ABSTRACT

Objectives: Previous studies suggested an inverse association between lipoprotein cholesterols and bleeding risk, while limited data was available about the predictive value of lipoproteins on intracerebral hemorrhage (ICH). Our recent research series showed that higher non-high-density lipoprotein cholesterol (non-HDLC) was an independent predictor of favourable 3-month outcome in ICH patients, we thus aimed to further investigate the association between non-HDLC levels and 1-year functional outcomes after ICH.

Design: Prospective multicenter cohort study.

Setting: 13 hospitals in Beijing, China.

Participants: A total of 666 ICH patients were included between December 2014 and September 2016.

Methods: Non-HDLC was calculated by subtracting HDL-C from TC**.** Patients were then grouped by non-HDLC levels into three categories: <3.4mmol/L, 3.4-4.2mmol/L, and ≥4.2mmol/L. Both the univariate and multivariate logistic regressions were used to assess the association between non-HDLC levels and 1-year unfavorable functional outcomes (modified Rankin Scale ≥3) in ICH patients. Moreover, sensitivity analysis was performed in ICH patients without statin use after admission.

Results: There were 33.5% (223/666) ICH patients identified with unfavorable functional outcomes at 1-year follow-up. In the univariate analysis, patients who achieved non-HDLC levels above 4.2 mmol/L had a 49% decreased risk of 1-year poor prognosis (OR 0.51, 95% CI 0.33-0.81). However, non-HDLC did not retain its independent prognostic value in multivariate analysis, the fully adjusted OR values were 1.00 (reference), 1.06 (0.63, 1.79), and 0.83 (0.45, 1.54) from the lowest to the highest non-HDLC group. Moreover, statin use after ICH onset made no difference to the long-term prognosis.

Conclusions: Non-HDLC was not an independent predictor for 1-year functional outcome in ICH patients,

For peer review only - http://bmjopen.bmj.com/site/about/guidelines.xhtml

irrespective of post-stroke statin use. The predictive value of well-recognized confounding factors was more dominant than non-HDLC on long-term prognosis.

Strengths and limitations of this study

- A multicenter, prospective, cohort study included 666 ICH patients from a total of 13 hospitals in Beijing.
- Our study filled the vacancy about the association between non-HDLC and 1-year functional outcomes, simultaneously shed light on the diverse impacts of non-HDLC on short-term and long-term prognosis in ICH patients.
- Sensitivity analysis was performed to evaluate the association between non-HDLC and 1-year functional outcomes in ICH patients with post-stroke statin use.
- Data regarding hematoma expansion and antithrombotic treatment were unavailable, further exploration is needed to verify our results.

For peer review only

For peer review only - http://bmjopen.bmj.com/site/about/guidelines.xhtml

INTRODUCTION

Intracerebral hemorrhage (ICH) is the second most common subtype of stroke, leading to severe disability and mortality.[1] Based on the nationally representative stroke survey in China published recently, ICH accounts for 25% of all strokes with an overall age-standardized incidence of 66.2 per 100,000 person-years.[2] Despite rapid advances in medicine, the management of ICH remains supportive without significant breakthroughs.[3] Approximately 30-48% of ICH patients died within one month in low- to middle-income countries and only 12-39% of survivors could achieve long-term functional independence.[1, 4]

The conventional view on lipid-lowering targets goes "the lower, the better" in patients with atherosclerotic cardiovascular disease. However, previous epidemiology studies suggested an inverse association between lipoprotein cholesterols and ICH risk, hematoma expansion, and mortality.[5, 6] Much remains to be discussed on the predictive value of lipoproteins on ICH. Our recent research series showed that low serum lipid levels were independent predictors of 3-month poor prognosis in ICH patients, and non-high-density lipoprotein cholesterol (non-HDLC) was the optimal parameter with high specificity.[7, 8] However, the literature has scant information regarding the association between non-HDLC and long-term ICH prognosis.

We thus aimed to investigate the association between serum non-HDLC levels and 1-year functional outcomes after ICH in this prospective cohort study.

METHODS

Study population

The study was conducted in accordance with the Declaration of Helsinki and was approved by

For peer review only - http://bmjopen.bmj.com/site/about/guidelines.xhtml

the Institutional Review Board of the Beijing Tiantan Hospital (KY2014-023-02). All participants or their legal representatives provided written informed consent.

Our study is a multicenter, prospective, cohort study conducted in a total of 13 hospitals, evaluating the medical quality of cerebral hemorrhage on different etiologies in Beijing. From December 2014 to September 2016, 1964 consecutive ICH patients agreed to participate in the study. A total of 1881 patients met the following inclusion criteria: (1) aged 18 years or older, (2) had their first CT scan done within 72h after symptom onset. After excluding 159 secondary ICH patients (caused by trauma, tumor, aneurysm, arteriovenous malformation, coagulopathy, or other causes) and 20 patients diagnosed as primary ventricular hemorrhage, 1702 patients with primary intraparenchymal hemorrhage were included. Moreover, 294 patients underwent surgical procedures (including craniotomy hematoma removal, hematoma puncture, extraventricular drainage, and so on), 15 patients with anticoagulant therapy before symptom onset, 588 patients with missing data on the non-HDLC level, and 139 patients lost to follow-up at 1-year were excluded. Eventually, 666 patients with spontaneous ICH from 13 sites were included (Figure 1).

Baseline information

Demographic information including age, sex, onset to admission time, past medical history (including hypertension, diabetes mellitus, hyperlipidemia, cerebral infarction, and ICH), personal habits (including smoking and drinking status), and medication history (including antiplatelet and statin therapy) of each patient was collected using a standard questionnaire at baseline. Neurological deficits were assessed using the National Institute of Health Stroke Scale (NIHSS)

For peer review only - http://bmjopen.bmj.com/site/about/guidelines.xhtml

and Glasgow Coma Scale (GCS) score by experienced neurologists on admission. Meanwhile, systolic and diastolic blood pressure (BP) were measured. A cranial CT scan was performed on admission and hematoma volume was then calculated as ABC/2 volumetric formula at each site.[9] The location of hematoma was further subdivided into supratentorial and infratentorial regions. ICH score was calculated based on five parameters, GCS score, ICH volume, the presence of intraventricular extension, location of hematoma, and age.[10]

Measurement of non-HDLC levels and other biochemical parameters

Blood samples were drawn from the antecubital vein the next morning after an overnight fast and analyzed within 4h. Total cholesterol (TC) was measured using the end-point test method and high-density lipoprotein cholesterol (HDL-C) was measured using a direct method. Non-HDLC was thus calculated by subtracting HDL-C from TC. Based on the National Lipid Association Recommendations,[11] non-HDLC levels were categorized into five groups: desirable, <3.4mmol/L; above desirable, 3.4-4.2mmol/L; borderline high, 4.2-5.0mmol/L; high, 5.0-5.8 mmol/L; and very high, ≥5.8 mmol/L. Accordingly, we integrated the last three groups into one group (≥4.2mmol/L) due to the limited number of patients.

For other biochemical parameters, random blood glucose was measured via the hexokinase/glucose-6-phosphate dehydrogenase method, serum creatinine was measured through rate reflectance spectrophotometry, white blood cell (WBC) together with platelet count were performed on EDTA with an ADVIA 120 counter (Siemens Healthcare Diagnostics, Saint-Denis, France).

For peer review only - http://bmjopen.bmj.com/site/about/guidelines.xhtml

Follow-up information and definition of 1-year ICH prognosis

Patients were followed up at 1-year after ICH onset via telephone interviews. Follow-up evaluation was performed by neurologists who were blinded to prognostic factors. 1-year prognosis of patients was evaluated by modified Ranking Scale (mRS) score and categorized as favorable (mRS<3) and unfavorable functional outcome groups (mRS≥3). Newly diagnosed stroke and the subtypes of stroke (both ischemic stroke and intracerebral hemorrhage) during the 1-year follow-up period were also documented.

Patient and public involvement

No patients were involved.

Statistical analysis

The patients were divided into three groups according to the clinical diagnosis of abnormal non-HDLC levels. Continuous variables were presented as median with interquartile range (IQR), categorical variables were described as count with percentage. The group differences of continuous variables were analyzed using ANOVA or Kruskal-Wallis test as appropriate, and for categorical variables, chi-squared tests were performed. Logistic regression was used to evaluate the association between non-HDLC levels and 1-year prognosis of ICH patients, with the lowest non-HDLC group (<3.4mmol/L) used as the reference. Both the univariate and multivariate analyses were conducted to estimate the odds ratios (ORs) and 95% confidence intervals (CIs). Kaplan-Meier curves were generated and the log-rank test was employed to perform comparisons between the non-HDLC levels. Cox proportional hazards regression analysis was

For peer review only - http://bmjopen.bmj.com/site/about/guidelines.xhtml

used to evaluate the risk of stroke and stroke subtypes, expressed as the hazard ratios (HRs) and 95% CIs. Multiple regression models were run as follows. Model 1 was adjusted for age and sex. Model 2 was adjusted for variates in model 1 plus premorbid mRS score (<3 or ≥3), history of ICH, glucose on admission, WBC on admission, baseline hematoma volume, hematoma location, time from onset to initial non-contrast CT, GCS score at admission, and systolic BP. *P*-values for trend were conducted using the three categories of non-HDLC as ordinal variables in the model. Additionally, sensitivity analysis was performed in ICH patients without statin use after admission (n=589). A 2-sided value of $p<0.05$ was considered statistically significant. All statistical analyses were performed using SAS software, version 9.4 (SAS Institute, Cary, NC, USA).

RESULTS

A total of 666 eligible patients were included, with a mean age of 59 years old (ranging from 51 to 68) and 69.1% (460/666) of them were males. Amongst them, 33.5% (223/666) were identified as 1-year poor outcomes, the proportion of which were 38.4%, 30.3%, and 24.2% from <3.4mmol/L group to ≥4.2mmol/L group.

Baseline characteristics

There were significant differences in age, prior statin use, diastolic BP, glucose on admission, WBC on admission, and statin use after admission among the three categories of non-HDLC levels ($p<0.05$, Table 1). Those with higher lipid levels were more likely to be younger, not a prior statin user, having higher diastolic BP and glucose on admission. While no statistical significance

For peer review only - http://bmjopen.bmj.com/site/about/guidelines.xhtml

was observed in sex, premorbid mRS scale, onset to admission time, past medical history, personal habits, prior antiplatelet use, NIHSS score, GCS score, SBP, creatinine, infections, time from onset to initial NCCT, hematoma volume, hematoma location, and ICH score between the three groups.

Table 1. Baseline characteristics of participants according to non-HDLC levels.

	Total	non-HDLC levels			*P*-value
		<3.4mmol/L	3.4-4.2mmol/L	⩾4.2mmol/L	
n (%)	666	359 (53.9)	175 (26.3)	132 (19.8)	
Age, years	59 (51, 68)	61 (53, 70)	57 (49, 67)	54 (48, 64)	<0.001
Male, n (%)	460 (69.1)	258 (71.9)	120 (68.6)	82 (62.1)	0.116
Onset to admission time, h	4.0 (1.8, 11.9)	3.8 (1.7, 11.1)	4.0 (2.0, 11.0)	4.0 (1.8, 14.7)	0.840
Premorbid mRS score					0.614
mRS<3	643 (96.5)	345 (96.1)	171 (97.7)	127 (96.2)	
mRS≥3	23 (3.5)	14 (3.9)	4 (2.3)	5 (3.8)	
Hypertension, n (%)	479 (71.9)	256 (71.3)	124 (70.9)	99 (75.0)	0.676
Diabetes mellitus, n (%)	106 (15.9)	55 (15.3)	29 (16.6)	22 (16.7)	0.902
Hyperlipidemia, n (%)	68 (10.2)	36 (10.0)	18 (10.3)	14 (10.6)	0.982
History of CI, n (%)	102 (15.3)	58 (16.2)	27 (15.4)	17 (12.9)	0.670
History of ICH, n (%)	20 (3.0)	15 (4.2)	3 (1.7)	2 (1.5)	0.141
Smoking, n (%)	223 (33.5)	127 (35.4)	57 (32.6)	39 (29.6)	0.458
Drinking, n (%)	256 (38.4)	139 (38.7)	69 (39.4)	48 (36.4)	0.850
Prior antiplatelet use, n (%)	110 (16.5)	61 (17.0)	28 (16.0)	21 (15.9)	0.771
Prior statin use, n (%)	44 (6.6)	31 (8.6)	10 (5.7)	3 (2.3)	0.036
NIHSS score on admission	8 (3, 13)	9 (3, 15)	7 (3, 13)	5 (2, 12)	0.083
GCS score on admission	14 (12, 15)	14 (12, 15)	15 (13, 15)	15 (13, 15)	0.063
SBP on admission, mmHg	160 (149, 183)	160 (150, 180)	160 (145, 183)	162 (150, 183)	0.564
DBP on admission, mmHg	95 (83, 105)	92 (80, 102)	96 (85, 106)	97 (85, 109)	0.024
Glucose on admission, mmol/L	6.9 (5.9, 8.4)	6.6 (5.8, 8.1)	7.0 (5.9, 8.6)	7.1 (6.0, 9.3)	0.032
WBC on admission, 10^9/L	8.4 (6.6, 10.9)	8.1 (6.3, 10.7)	9.1 (7.0, 11.7)	7.1 (6.0, 9.3)	0.007
Platelets on admission, 10^9/L	212 (175, 252)	202 (164, 238)	218 (180, 259)	230 (192, 265)	<0.001
Creatinine on admission, μmol/L	64.0 (53.0, 77.3)	64.6 (54.0, 76.4)	65.0 (52.3, 79.0)	62.0 (50.1, 76.0)	0.223
Statin use after admission, n (%)	77 (11.6)	19 (5.3)	30 (17.1)	28 (21.2)	<0.001
Infections, n (%)	136 (20.4)	77 (21.5)	39 (22.3)	20 (15.2)	0.239
Time from onset to initial NCCT, h	5.2 (2.3, 16.7)	5.2 (2.2, 14.8)	5.1 (2.3, 19.6)	4.8 (2.3, 19.4)	0.738
Baseline hematoma volume, ml	10.5 (5.0, 23.4)	10.7 (5.0, 25.0)	10.4 (5.5, 23.1)	10.0 (4.9, 16.8)	0.379
Hematoma location					0.251
Supratentorial, n (%)	599 (89.7)	327 (91.2)	155 (88.2)	117 (87.5)	
Infratentorial, n (%)	67 (10.3)	31 (8.8)	23 (11.8)	16 (12.5)	
Secondary ventricular hemorrhage	181 (27.2)	100 (27.9)	43 (24.6)	38 (28.8)	0.652
ICH score	0 (0, 1)	0 (0, 1)	0 (0, 1)	0 (0, 1)	0.447

Values are (%) for categorical variables and median (IQR) for continuous variables.

mRS, modified Rankin Scale; CI, cerebral infarction; ICH, intracerebral hemorrhage; NIHSS, National Institutes of Health Stroke Scale; GCS, Glasgow Coma Scale; SBP, systolic blood

For peer review only - http://bmjopen.bmj.com/site/about/guidelines.xhtml

pressure; DBP, diastolic blood pressure; WBC, white blood cells; NCCT, non-contrast CT.

Correlation between baseline non-HDLC and 1-year prognosis in ICH patients

In the univariate analysis, higher non-HDLC levels were significantly associated with decreased risk of 1-year poor outcome (*p*=0.002). Patients who achieved non-HDLC above 4.2mmol/L had a 49% lower risk of poor functional outcome at 1 year (OR 0.51, 95% CI 0.33-0.81). While no statistical difference was retained after adjusting for age, sex, and potential confounding factors (*p*>0.05). In the fully adjusted model (Model 2), the OR values were 1.00 (reference), 1.06 (0.63, 1.79), and 0.83 (0.45, 1.54) from the lowest to the highest non-HDLC group. Moreover, the results maintained consistency in sensitivity analysis among patients without statin use after admission (*p*=0.842, Table 2).

Table 2. Odds ratios and 95% CI for 1-year poor outcome (mRS ≥3) according to non-HDLC levels.

	non-HDLC levels			Continuous scale	*P* for trend
	<3.4mmol/L	3.4-4.2mmol/L	≥4.2mmol/L		
1-year poor outcome, n (%)	138 (38.4)	53 (30.3)	32 (24.2)		
Univariate analysis	Ref.	0.70 (0.47, 1.02)	0.51 (0.33, 0.81)	0.71 (0.58, 0.88)	0.002
Multivariate analysis					
Model 1	Ref.	0.82 (0.54, 1.23)	0.66 (0.41, 1.06)	0.81 (0.65, 1.02)	0.075
Model 2	Ref.	1.06 (0.63, 1.79)	0.83 (0.45, 1.54)	0.89 (0.76, 1.05)	0.694
Sensitivity analysis	Ref.	0.92 (0.53, 1.61)	1.12 (0.58, 2.16)	0.92 (0.78, 1.08)	0.842

Data are OR (95% CI) unless otherwise stated.
Model 1 adjusted for age and sex.
Model 2 adjusted for variates in model 1 plus premorbid mRS score (<3 or ≥3), history of ICH, glucose on admission, WBC on admission, baseline hematoma volume, hematoma location, time from onset to initial non-contrast CT, GCS score at admission, systolic blood pressure.
Sensitivity analysis was performed in ICH patients without statin use after admission (n=589), and adjusted for variates in model 2.

Notably, age, premorbid mRS score (<3 or ≥3), and baseline hematoma volume were positively associated with 1-year poor prognosis in the multivariate analysis. Whereas, higher GCS score at

For peer review only - http://bmjopen.bmj.com/site/about/guidelines.xhtml

admission was an independent predictor of favorable outcomes. Additional detailed information was given in Figure 2.

In the process of statistics, we also calculated the association between the quartiles of non-HDLC with 1-year poor outcome (data was shown in Supplementary Table 1). The highest quartile of non-HDLC was significantly associated with decreased risk of 1-year poor outcome, while no statistical difference was retained after adjusting for confounding factors. Due to the identical results of the two cut-off methods, we thus chose the risk-stratified levels of non-HDLC which had more instructive clinical significance.

Correlation between baseline non-HDLC and stroke risk

We further investigated the correlation between non-HDLC levels and another stroke (ischemic or hemorrhagic) risk. In univariate analysis, the cumulative incidences of total stroke, ischemic stroke, and ICH were not statistically different among non-HDLC levels (log-rank test, all $P > 0.05$, Figure 3). In multivariate analysis, no correlation was identified between the three groups either (Table 3). When the quartile of non-HDLC was set as the cut-off, similar negative results were also obtained (data was not shown).

Table 3. Hazard ratios for stroke according to non-HDLC levels.

	non-HDLC levels			*P* for trend	Per 1 SD increase
	<3.4mmol/L	3.4-4.2mmol/L	≥4.2mmol/L		
**Total stroke**					
Events, n (%)	10 (2.8)	6 (3.4)	3 (2.3)		
Model 1	Ref.	1.06 (0.38, 2.94)	0.71 (0.19, 2.61)	0.88 (0.49, 1.59)	0.96 (0.67, 1.39)
Model 2	Ref.	1.44 (0.50, 4.22)	0.83 (0.21, 3.25)	0.98 (0.54, 1.80)	1.00 (0.74, 1.35)
**Ischemic stroke**					
Events, n (%)	6 (1.7)	2 (1.1)	2 (1.5)		

For peer review only - http://bmjopen.bmj.com/site/about/guidelines.xhtml

For peer review only

Model 1	Ref.	0.56 (0.11, 2.79)	0.75 (0.15, 3.79)	0.81 (0.35, 1.86)	0.94 (0.61, 1.47)
Model 2	Ref.	0.73 (0.14, 3.89)	0.65 (0.12, 3.67)	0.80 (0.34, 1.86)	0.99 (0.75, 1.32)
**Intracerebral hemorrhage**					
Events, n (%)	4 (1.1)	4 (2.3)	2 (1.5)		
Model 1	Ref.	1.86 (0.46, 7.52)	1.24 (0.22, 6.89)	1.18 (0.55, 2.54)	1.01 (0.53, 1.94)
Model 2	Ref.	2.84 (0.61, 13.14)	1.80 (0.28, 11.53)	1.41 (0.63, 3.19)	1.07 (0.52, 2.21)

Data are HR (95% CI) unless otherwise stated.

Model 1 adjusted for age and sex.

Model 2 adjusted for variates in model 1 plus prior mRS scale (<3 or ≥3) history of ICH, glucose on admission, WBC on admission, baseline hematoma volume, hematoma location, time from onset to initial non-contrast CT, GCS score at admission, systolic blood pressure.

For peer review only

DISCUSSION

This study provided evidence on the association between non-HDLC levels and long-term functional outcomes in ICH patients. Although non-HDLC was a significant 1-year predictor in univariate analysis, it did not retain its independent prognostic value in multivariate analysis. Moreover, statin use after ICH onset made no difference to the long-term prognosis.

In our study, the prevalence of 1-year functional independence in ICH patients was 66.5% (443/666), far outweighing the data previously reported.[4] According to the inclusion and exclusion criteria, severe cases who underwent surgical treatment or lost to follow-up were not enrolled. It is noteworthy that per 1 mmol/L increment in non-HDLC yielded a 29% decreased risk of 1-year poor prognosis (crude OR 0.71, 95% CI 0.58-0.88). However, contrary to our previous research finding of the independent role of non-HDLC on short-term functional outcomes,[7] the results of this study showed that age, premorbid mRS score, baseline hematoma volume, admission GCS score, rather than non-HDLC level, were independent predictors for long-term functional outcomes in ICH patients. The validated predictors mentioned above kept high conformity with the items in ICH Functional Outcome Score, an effective prognostic model for 1-year poor functional outcomes after ICH,[12] whereas the absolute magnitude effect of low non-

For peer review only - http://bmjopen.bmj.com/site/about/guidelines.xhtml

HDLC level on ICH prognosis was likely to be small and overshadowed with time. Beyond that, the amount of rehabilitation with functional gains might also be related.[13]

It was reported that low levels of LDL-C and TC were associated with hematoma expansion.[14, 15] As containing all the atherogenic lipoproteins, non-HDLC was served as the preferred target of lipid-lowering therapy.[16] The potential mechanisms regarding the association between hypolipidemia and hematoma expansion, including impaired endothelial integrity,[17] necrotic medial smooth muscle cells,[18] increased erythrocyte fragility,[19] inhibited platelet aggregation,[20] and the resultant incident cerebral microbleeds.[21] Despite the theoretical basis, our study failed to show an independent correlation between non-HDLC levels and 1-year functional outcomes in ICH patients. The secondary injury caused by low levels of lipoproteins in ICH patients was associated with short-term prognosis (30-day, 3-month),[22, 23] while its impact on long-term prognosis (1-year) was negative, which merits further investigation due to the limited sample size and incomplete neuroimaging data on hematoma expansion in our study.

Statin treatment is another major concern,[24] there were respectively 6.6% (44/666) and 11.6% (77/666) patients with pre- and post-stroke statin use in our study. Two recent meta-analyses concluded that there was no evidence to suggest pre-stroke statin therapy may increase bleeding risk in the context of ICH.[25, 26] Whether to start, continue, or stop statin treatment in ICH patients has aroused great concern, we thus conducted a sensitivity analysis to evaluate the effect of statin exposure after admission on ICH prognosis. No significant difference was detected between non-HDLC levels and 1-year prognosis in ICH patients in our study, irrespective of post-stroke statin use. A recent review indicated that statin should be applied after weighing the pros and cons given its pleiotropic as well as lipid-lowering effects.[27] Because of the

For peer review only - http://bmjopen.bmj.com/site/about/guidelines.xhtml

relatively low stain exposure rate in our study, it is necessary to conduct randomized controlled trials around this topic.

Our study filled the vacancy about the association between non-HDLC and 1-year functional outcomes, simultaneously shed light on the diverse impacts of non-HDLC on short-term and long-term prognosis in ICH patients. Nonetheless, there are still some limitations. First, the follow-up radiological information was unavailable, which makes it difficult to verify the intermediate role of hematoma expansion between non-HDLC and poor prognosis. Secondly, ICH caused by cerebral amyloid angiopathy has a higher rebleeding risk than hypertensive one,[28] while data regarding the cause of ICH was not documented in our study. Despite no correlation was observed between the history of ICH and 1-year functional outcome, the impact of ICH etiology merits further investigation. Thirdly, medication therapy regarding antiplatelet or anticoagulation agents were not included in the multivariate analysis, whereas accumulating researches proved that antithrombotic treatment increased the risk of cerebral microbleeds as well as future ICH.[29, 30] Although we collected pre-ictus antiplatelet use, restricted by the small sample size, further research is needed to provide insight into the relationship. Moreover, since our study based on a highly selected population with small hematoma and relatively good neurologic status to achieve precise research, the findings cannot be generalized to the whole ICH population.

CONCLUSION

In conclusion, non-HDLC was not an independent predictor for 1-year functional outcome in ICH patients, irrespective of post-stroke statin use. The predictive value of well-recognized

For peer review only - http://bmjopen.bmj.com/site/about/guidelines.xhtml

confounding factors was more dominant than non-HDLC on long-term poor prognosis. Further prospective studies are needed to assess the impact of lower non-HLDC levels on ICH prognosis.

Author affiliations

1 China National Clinical Research Center for Neurological Diseases, Beijing, China

2 Department of Neurology, Beijing Tiantan Hospital, Capital Medical University, Beijing, China

3 Research Unit of Artificial Intelligence in Cerebrovascular Disease, Chinese Academy of Medical Sciences, Beijing, China

Contributors YW and JW performed the experiments, interpreted the results of statistical analysis, and drafted the manuscript. AW conducted the statistical analysis and interpreted the data. RJ revised the manuscript for intellectual content. WW and XZ had full access to all of the data and take responsibility for the integrity of the data and the accuracy of the data analysis.

Funding Our study was supported by grants from the Chinese Academy of Medical Sciences Innovation Fund for Medical Sciences (2019-I2M-5-029), Beijing Natural Science Foundation (Z200016), Beijing Municipal Committee of Science and Technology (Z201100005620010), and Ministry of Science and Technology of the People's Republic of China (2018YFC1705003).

Competing interests None declared.

Patient consent for publication Not applicable.

Ethics approval The study was approved by the Central Institutional Review Board of Beijing

For peer review only - http://bmjopen.bmj.com/site/about/guidelines.xhtml

For peer review only

Tiantan Hospital (KY2014-023-02) and written informed consent was obtained.

Provenance and peer review Not commissioned; externally peer reviewed.

Data sharing statement Some or all datasets generated during and/or analyzed during the current study are not publicly available but are available from the corresponding author on reasonable request.

For peer review only

References

1. An SJ, Kim TJ, Yoon BW. Epidemiology, Risk Factors, and Clinical Features of Intracerebral Hemorrhage: An Update. J Stroke 2017;19:3-10.
2. Wang W, Jiang B, Sun H, et al. Prevalence, Incidence, and Mortality of Stroke in China: Results from a Nationwide Population-Based Survey of 480 687 Adults. Circulation 2017;135:759-771.
3. Hemphill JC, 3rd, Greenberg SM, Anderson CS, et al. Guidelines for the Management of Spontaneous Intracerebral Hemorrhage: A Guideline for Healthcare Professionals From the American Heart Association/American Stroke Association. Stroke 2015;46:2032-2060.
4. Feigin VL, Lawes CM, Bennett DA, Barker-Collo SL, Parag V. Worldwide stroke incidence and early case fatality reported in 56 population-based studies: a systematic review. Lancet Neurol 2009;8:355-369.
5. Wang X, Dong Y, Qi X, Huang C, Hou L. Cholesterol levels and risk of hemorrhagic stroke: a systematic review and meta-analysis. Stroke 2013;44:1833-1839.
6. Chang JJ, Katsanos AH, Khorchid Y, et al. Higher low-density lipoprotein cholesterol levels are associated with decreased mortality in patients with intracerebral hemorrhage. Atherosclerosis 2018;269:14-20.
7. Feng H, Wang X, Wang W, Zhao X. Association Between Non-high-density Lipoprotein Cholesterol and 3-Month Prognosis in Patients With Spontaneous Intracerebral Hemorrhage. Front Neurol 2020;11:920.
8. Feng H, Wang X, Wang W, Zhao X. Lipid Levels and 3-Month Prognosis After Spontaneous Intracerebral Hemorrhage in Women. Front Neurol 2021;12:690194.
9. Kothari RU, Brott T, Broderick JP, et al. The ABCs of measuring intracerebral hemorrhage volumes. Stroke 1996;27:1304-1305.
10. Hemphill JC, 3rd, Bonovich DC, Besmertis L, Manley GT, Johnston SC. The ICH score: a simple, reliable grading scale for intracerebral hemorrhage. Stroke 2001;32:891-897.
11. Jacobson TA, Ito MK, Maki KC, et al. National lipid association recommendations for patient-centered management of dyslipidemia: part 1--full report. J Clin Lipidol 2015;9:129-169.
12. Ji R, Shen H, Pan Y, et al. A novel risk score to predict 1-year functional outcome after intracerebral hemorrhage and comparison with existing scores. Crit Care 2013;17:R275.
13. Kamo T, Momosaki R, Suzuki K, et al. Effectiveness of Intensive Rehabilitation Therapy on Functional Outcomes After Stroke: A Propensity Score Analysis Based on Japan Rehabilitation

For peer review only - http://bmjopen.bmj.com/site/about/guidelines.xhtml

Database. J Stroke Cerebrovasc Dis 2019;28:2537-2542.

14. Rodriguez-Luna D, Rubiera M, Ribo M, et al. Serum low-density lipoprotein cholesterol level predicts hematoma growth and clinical outcome after acute intracerebral hemorrhage. Stroke 2011;42:2447-2452.
15. Broderick JP, Diringer MN, Hill MD, et al. Determinants of intracerebral hemorrhage growth: an exploratory analysis. Stroke 2007;38:1072-1075.
16. National Cholesterol Education Program Coordinating Committee. Third Report of the National Cholesterol Education Program (NCEP) Expert Panel on Detection, Evaluation, and Treatment of High Blood Cholesterol in Adults (Adult Treatment Panel III) final report. Circulation 2002;106:3143-3421.
17. Bang OY, Saver JL, Liebeskind DS, et al. Cholesterol level and symptomatic hemorrhagic transformation after ischemic stroke thrombolysis. Neurology 2007;68:737-742.
18. Ooneda G, Yoshida Y, Suzuki K, et al. Smooth muscle cells in the development of plasmatic arterionecrosis, arteriosclerosis, and arterial contraction. Blood Vessels 1978;15:148-156.
19. Yamori Y, Nara Y, Horie R, Ooshima A. Abnormal membrane characteristics of erythrocytes in rat models and men with predisposition to stroke. Clin Exp Hypertens 1980;2:1009-1021.
20. Chui DH, Marotta F, Rao ML, Liu DS, Zhang SC, Ideo C. Cholesterol-rich LDL perfused at physiological LDL-cholesterol concentration induces platelet aggregation and PAF-acetylhydrolase activation. Biomed Pharmacother 1991;45:37-42.
21. Lee SH, Bae HJ, Yoon BW, Kim H, Kim DE, Roh JK. Low concentration of serum total cholesterol is associated with multifocal signal loss lesions on gradient-echo magnetic resonance imaging: analysis of risk factors for multifocal signal loss lesions. Stroke 2002;33:2845-2849.
22. Rodriguez-Luna D, Rubiera M, Ribo M, et al. Ultraearly hematoma growth predicts poor outcome after acute intracerebral hemorrhage. Neurology 2011;77:1599-1604.
23. Sembolini A, Romoli M, Pannacci U, et al. Acute hematoma expansion after spontaneous intracerebral hemorrhage: risk factors and impact on long-term prognosis. Neurol Sci 2020;41:2503-2509.
24. Goldstein LB, Amarenco P, Szarek M, et al. Hemorrhagic stroke in the Stroke Prevention by Aggressive Reduction in Cholesterol Levels study. Neurology 2008;70:2364-2370.
25. Hackam DG, Woodward M, Newby LK, et al. Statins and intracerebral hemorrhage: collaborative systematic review and meta-analysis. Circulation 2011;124:2233-2242.
26. Ziff OJ, Banerjee G, Ambler G, Werring DJ. Statins and the risk of intracerebral haemorrhage in patients with stroke: systematic review and meta-analysis. J Neurol Neurosurg Psychiatry 2019;90:75-83.
27. Endres M, Nolte CH, Scheitz JF. Statin Treatment in Patients With Intracerebral Hemorrhage. Stroke 2018;49:240-246.
28. Pasi M, Charidimou A, Boulouis G, et al. Mixed-location cerebral hemorrhage/microbleeds: Underlying microangiopathy and recurrence risk. Neurology 2018;90:e119-e126.
29. Qiu J, Ye H, Wang J, Yan J, Wang J, Wang Y. Antiplatelet Therapy, Cerebral Microbleeds, and Intracerebral Hemorrhage: A Meta-Analysis. Stroke 2018;49:1751-1754.
30. Lioutas VA, Goyal N, Katsanos AH, et al. Optimization of risk stratification for anticoagulation-associated intracerebral hemorrhage: net risk estimation. J Neurol 2020;267:1053-1062.

For peer review only - http://bmjopen.bmj.com/site/about/guidelines.xhtml

Figure Legends

Figure 1. Flow chart for selection of study participants.

ICH, intracerebral hemorrhage; non-HDLC, non-high-density lipoprotein cholesterol.

Figure 2. Multivariate predictors of 1-year poor outcome among ICH patients.

Non-HDLC, non-high-density lipoprotein cholesterol; ICH, intracerebral hemorrhage; mRS, modified Rankin Scale; WBC, white blood cells; NCCT, non-contrast CT; GCS, Glasgow Coma Scale; SBP, systolic blood pressure.

Figure 3. Cumulative incidences of (A) total stroke, (B) ischemic stroke, and (C) intracerebral hemorrhage according to non-HDLC levels.

Non-HDLC, non-high-density lipoprotein cholesterol.

For peer review only

For peer review only - http://bmjopen.bmj.com/site/about/guidelines.xhtml

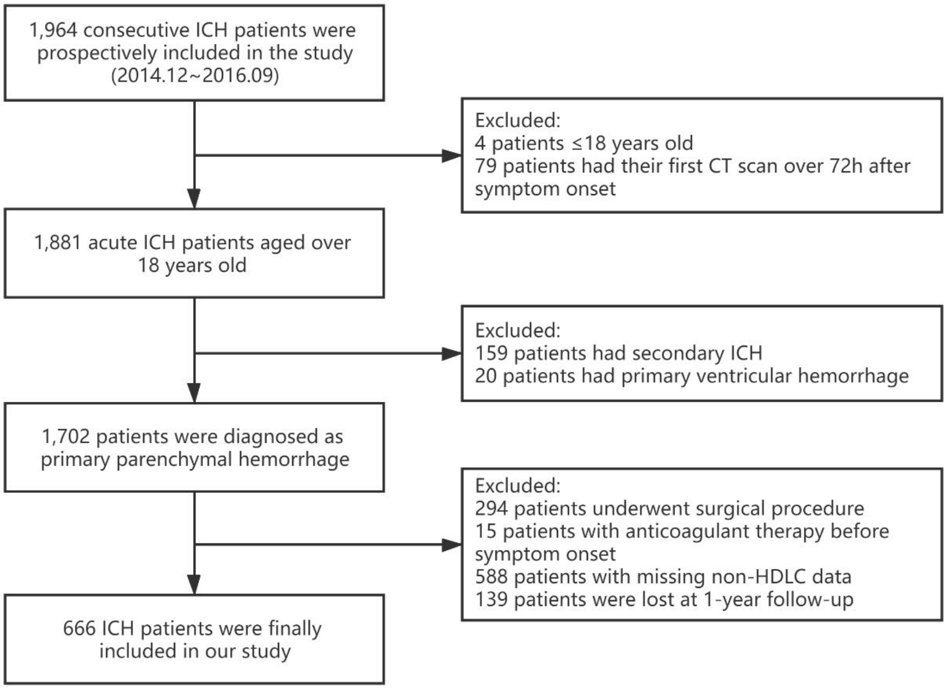

Figure 1. Flow chart for selection of study participants.
ICH, intracerebral hemorrhage; non-HDLC, non-high-density lipoprotein cholesterol.

146x108mm (220 x 220 DPI)

Variables	Poor outcome (mRS ≥3, n=178)	Odds ratio (95% CI)
non-HDLC (vs <3.4mmol/L)		
3.4-4.2mmol/L	1.06 (0.63, 1.79)	
≥4.2mmol/L	0.83 (0.45, 1.54)	
Age, years	1.08 (1.06, 1.10)	
Male	0.84 (0.52, 1.36)	
Premorbid mRS score ≥3	3.89 (1.43, 6.77)	
History of ICH	2.80 (0.95, 5.27)	
Glucose on admission, mmol/L	1.02 (0.95, 1.11)	
WBC on admission, 10E9/L	1.00 (0.93, 1.08)	
Platelet on admission, 10E9/L	1.00 (0.99, 1.00)	
Baseline hematoma volume, mL	1.02 (1.01, 1.04)	
Infratentorial hemorrhage	0.82 (0.37, 1.82)	
Tme from onset to initial NCCT, hours	0.99 (0.98, 1.01)	
GCS score on admission	0.73 (0.66, 0.80)	
SBP, mmHg	0.99 (0.99, 1.00)	

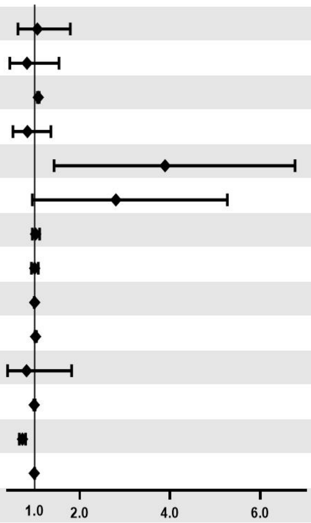

Figure 2. Multivariate predictors of 1-year poor outcome among ICH patients.
Non-HDLC, non-high-density lipoprotein cholesterol; ICH, intracerebral hemorrhage; mRS, modified Rankin Scale; WBC, white blood cells; NCCT, non-contrast CT; GCS, Glasgow Coma Scale; SBP, systolic blood pressure.

184x114mm (192 x 192 DPI)

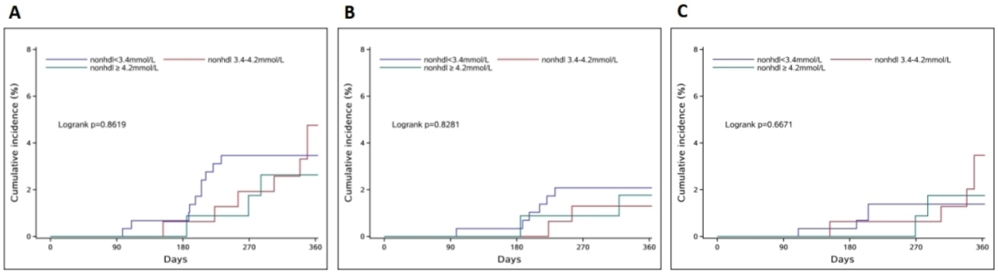

Figure 3. Cumulative incidences of (A) total stroke, (B) ischemic stroke, and (C) intracerebral hemorrhage according to non-HDLC levels.

185x52mm (192 x 192 DPI)

Supplementary Table 1. Odds ratios and 95% CI for 1-year poor outcome (mRS ≥3) according to non-HDLC quartiles.

	non-HDLC quartiles				Continuous scale	*P* for trend
	Q1	Q2	Q3	Q4		
1-year poor outcome, n (%)	71 (43.3)	58 (34.5)	54 (32.3)	40 (24.0)		
Univariate analysis	Ref.	0.69 (0.44, 1.08)	0.63 (0.40, 0.98)	0.41 (0.26, 0.66)	0.76 (0.66, 0.88)	<0.001
Multivariate analysis						
Model 1	Ref.	0.80 (0.50, 1.29)	0.84 (0.52, 1.36)	0.57 (0.35, 0.95)	0.85 (0.73, 1.00)	0.049
Model 2	Ref.	0.81 (0.44, 1.50)	1.03 (0.56, 1.90)	0.71 (0.37, 1.37)	0.93 (0.76, 1.14)	0.468
Sensitivity analysis	Ref.	0.83 (0.43, 1.60)	1.14 (0.60, 2.18)	0.76 (0.39, 1.51)	0.96 (0.77, 1.18)	0.673

Data are OR (95% CI) unless otherwise stated.

Model 1 adjusted for age and sex.

Model 2 adjusted for variates in model 1 plus prior mRS scale (<3 or ≥3) history of ICH, glucose on admission, WBC on admission, baseline hematoma volume, hematoma location, time from onset to initial non-contrast CT, GCS score at admission, systolic blood pressure.

Sensitivity analysis was performed in ICH patients without statin use after admission (n=589), and adjusted for variates in model 2.

For peer review only

STROBE Statement—Checklist of items that should be included in reports of ***cohort studies***

	Item No	Recommendation	Page No
**Title and abstract**	1	(*a*) Indicate the study's design with a commonly used term in the title or the abstract	1
		(*b*) Provide in the abstract an informative and balanced summary of what was done and what was found	2
**Introduction**			
Background/rationale	2	Explain the scientific background and rationale for the investigation being reported	4
Objectives	3	State specific objectives, including any prespecified hypotheses	4
**Methods**			
Study design	4	Present key elements of study design early in the paper	5
Setting	5	Describe the setting, locations, and relevant dates, including periods of recruitment, exposure, follow-up, and data collection	5-6
Participants	6	(*a*) Give the eligibility criteria, and the sources and methods of selection of participants. Describe methods of follow-up	5,7
		(*b*) For matched studies, give matching criteria and number of exposed and unexposed	NA
Variables	7	Clearly define all outcomes, exposures, predictors, potential confounders, and effect modifiers. Give diagnostic criteria, if applicable	7
Data sources/ measurement	8*	For each variable of interest, give sources of data and details of methods of assessment (measurement). Describe comparability of assessment methods if there is more than one group	5-6
Bias	9	Describe any efforts to address potential sources of bias	7
Study size	10	Explain how the study size was arrived at	5
Quantitative variables	11	Explain how quantitative variables were handled in the analyses. If applicable, describe which groupings were chosen and why	7
Statistical methods	12	(*a*) Describe all statistical methods, including those used to control for confounding	7
		(*b*) Describe any methods used to examine subgroups and interactions	7
		(*c*) Explain how missing data were addressed	NA
		(*d*) If applicable, explain how loss to follow-up was addressed	NA
		(*e*) Describe any sensitivity analyses	7
**Results**			
Participants	13*	(a) Report numbers of individuals at each stage of study—eg numbers potentially eligible, examined for eligibility, confirmed eligible, included in the study, completing follow-up, and analysed	5
		(b) Give reasons for non-participation at each stage	5
		(c) Consider use of a flow diagram	5
Descriptive data	14*	(a) Give characteristics of study participants (eg demographic, clinical, social) and information on exposures and potential confounders	8
		(b) Indicate number of participants with missing data for each variable of interest	5
		(c) Summarise follow-up time (eg, average and total amount)	NA
Outcome data	15*	Report numbers of outcome events or summary measures over time	8

For peer review only

Main results	16	(*a*) Give unadjusted estimates and, if applicable, confounder-adjusted estimates and their precision (eg, 95% confidence interval). Make clear which confounders were adjusted for and why they were included	9-10
		(*b*) Report category boundaries when continuous variables were categorized	9-10
		(*c*) If relevant, consider translating estimates of relative risk into absolute risk for a meaningful time period	NA
Other analyses	17	Report other analyses done—eg analyses of subgroups and interactions, and sensitivity analyses	9
**Discussion**			
Key results	18	Summarise key results with reference to study objectives	10
Limitations	19	Discuss limitations of the study, taking into account sources of potential bias or imprecision. Discuss both direction and magnitude of any potential bias	12
Interpretation	20	Give a cautious overall interpretation of results considering objectives, limitations, multiplicity of analyses, results from similar studies, and other relevant evidence	10-11
Generalisability	21	Discuss the generalisability (external validity) of the study results	11
**Other information**			
Funding	22	Give the source of funding and the role of the funders for the present study and, if applicable, for the original study on which the present article is based	13

*Give information separately for exposed and unexposed groups.

Note: An Explanation and Elaboration article discusses each checklist item and gives methodological background and published examples of transparent reporting. The STROBE checklist is best used in conjunction with this article (freely available on the Web sites of PLoS Medicine at http://www.plosmedicine.org/, Annals of Internal Medicine at http://www.annals.org/, and Epidemiology at http://www.epidem.com/). Information on the STROBE Initiative is available at http://www.strobe-statement.org.

For peer review only

For peer review only - http://bmjopen.bmj.com/site/about/guidelines.xhtml
